# Neonate personality affects early-life resource acquisition in a large social mammal

**DOI:** 10.1093/beheco/arac072

**Published:** 2022-08-09

**Authors:** Bawan Amin, Dómhnall J Jennings, Alison Norman, Andrew Ryan, Vasiliki Ioannidis, Alice Magee, Hayley-Anne Haughey, Amy Haigh, Simone Ciuti

**Affiliations:** Laboratory of Wildlife Ecology and Behaviour, School of Biology and Environmental Science, University College Dublin, Dublin, Ireland; School of Biological Sciences, Queen’s University Belfast, Belfast, UK; Laboratory of Wildlife Ecology and Behaviour, School of Biology and Environmental Science, University College Dublin, Dublin, Ireland; Laboratory of Wildlife Ecology and Behaviour, School of Biology and Environmental Science, University College Dublin, Dublin, Ireland; Laboratory of Wildlife Ecology and Behaviour, School of Biology and Environmental Science, University College Dublin, Dublin, Ireland; Laboratory of Wildlife Ecology and Behaviour, School of Biology and Environmental Science, University College Dublin, Dublin, Ireland; Laboratory of Wildlife Ecology and Behaviour, School of Biology and Environmental Science, University College Dublin, Dublin, Ireland; Laboratory of Wildlife Ecology and Behaviour, School of Biology and Environmental Science, University College Dublin, Dublin, Ireland; Laboratory of Wildlife Ecology and Behaviour, School of Biology and Environmental Science, University College Dublin, Dublin, Ireland

**Keywords:** anti-predator, fallow deer, foraging, juvenile, temperament, vigilance

## Abstract

Although it is widely acknowledged that animal personality plays a key role in ecology, current debate focuses on the exact role of personality in mediating life-history trade-offs. Crucial for our understanding is the relationship between personality and resource acquisition, which is poorly understood, especially during early stages of development. Here we studied how among-individual differences in behavior develop over the first 6 months of life, and their potential association with resource acquisition in a free-ranging population of fallow deer (*Dama dama*). We related neonate physiological (heart rate) and behavioral (latency to leave at release) anti-predator responses to human handling to the proportion of time fawns spent scanning during their first summer and autumn of life. We then investigated whether there was a trade-off between scanning time and foraging time in these juveniles, and how it developed over their first 6 months of life. We found that neonates with longer latencies at capture (i.e., risk-takers) spent less time scanning their environment, but that this relationship was only present when fawns were 3–6 months old during autumn, and not when fawns were only 1–2 months old during summer. We also found that time spent scanning was negatively related to time spent foraging and that this relationship became stronger over time, as fawns gradually switch from a nutrition rich (milk) to a nutrition poor (grass) diet. Our results highlight a potential mechanistic pathway in which neonate personality may drive differences in early-life resource acquisition of a large social mammal.

## Teaser

There is an ever-increasing interest in behavioral differences between individuals, or “animal personality”. Here we show how these differences develop over the first six months in fawns of a fallow deer population. Individuals that behave boldly as newborns when captured by humans still display bold behavior months later, which may allow them to acquire more resources. These findings shed light on the role that personality plays in ecology and evolution early in life.

## INTRODUCTION

Consistent among-individual differences (i.e., “animal personality”) play an important role in ecology and evolution (Dall et al. 2012; Wolf and Weissing 2012). More than a decade ago, behavioral ecologists aimed at incorporating animal personality into life-history theory. One of the most prominent hypotheses formed from that attempt is the extended pace-of-life syndrome hypothesis (POLS), which suggests that among-individual differences in behavior mediate within-species differences in life-history strategies on a fast-slow continuum (Réale et al. 2010; Dammhahn et al. 2018). Within this framework, behavior, physiology, and life-history are expected to covary. Individuals with a faster pace-of-life (POL) are expected to be bolder, more active, and to allocate more resources to growth and short-term reproduction than individuals with a slower POL (Réale et al. 2010). This increased resource allocation in current growth or reproduction is predicted to trade-off against survival: animals with a faster POL are expected to have a shorter lifespan relative to those with a slower POL (Réale et al. 2010; Dammhahn et al. 2018).

Empirical research has, however, not provided conclusive results for the main predictions of POLS (Royauté et al. 2018; Moiron et al. 2020; Laskowski et al. 2021), suggesting that there is a greater complexity than expected in the covariation between behavior and life-history. In particular, recent meta-analyses have failed to confirm the relationships between personality and survival as predicted under POLS (Royaute et al. 2018; Moiron et al. 2020; Haave-Audet et al, 2022). Moiron et al. (2020) found no evidence that bolder individuals paid a survival cost, and even found some evidence that bolder individuals survive for longer in wild populations, whereas Haave-Audet et al. (2022) found that behaviors that were associated with higher reproduction were also associated with higher survival. This lack of trade-offs between life-history traits indicates that there is little variation among individuals in resource allocation, but that there is rather significant variation in the amount of resources among individuals (Van Noordwijk and De Jong 1986). Personality could play a key role in maintaining these resource differences among individuals, if among-individual variation in behavior is closely related to resource acquisition, with bolder animals gaining more resources than shyer ones. In that case, the trade-off between survival, current growth, and reproduction may be weakened or even disappear at the among-individual level (Laskowski et al. 2021). It is therefore necessary to gain a better understanding of how personality relates to resource acquisition, in order to gain a better insight into the interactions between personality and life-history.

Additional complexity regarding contrasting evidence in animal personality studies is associated with changes of behavior over different life-stages (Stamps and Groothuis 2010; Cabrera et al. 2021). Although there is evidence that individuals behave in a consistent way within life-stages, including the earliest stages of life (Fucikova et al. 2009; Guenther and Trillmich 2015; Amin et al. 2016; Dhellemmes et al. 2020), the same cannot be said of individual consistency across different life-stages (see Cabrera et al. 2021 for a review and references therein). Furthermore, the limited number of studies that have investigated among-individual differences across life-stages have either done so on captive populations (Wuerz and Krüger 2015; Favati et al. 2016; Neave et al. 2020), or have measured behavior only during capture (Petelle et al. 2013; Class and Brommer 2015) or within artificial settings (Hall et al. 2015; Kelley et al. 2015). There is a paucity of studies that have investigated whether these traits measured in controlled settings are actually related to life-history in the wild (Niemelä and Dingemanse 2014).

Consequently, the relationship between animal personality and life-history related traits during early stages of development in a wild setting has yet to be tested. We therefore tested whether repeatable among-individual differences were associated with behavioral strategies affecting early-life resource acquisition in a free-living population of fallow deer fawns (*Dama dama*). These were monitored from birth to 6 months, through the key transition from solitary to group-living life. Fallow deer are a hider species (Lent 1974): fawns experience a relatively solitary life during their first 2–4 weeks of life remaining hidden in vegetation while occasionally being visited by their mother for maternal care (Chapman and Chapman 1997). We recently showed that repeatable among-individual differences are present during the first days of life in this population (Amin et al. 2021). Some neonates display repeatable active responses—i.e., elevated heart rates and short latency to leave when released—whereas other neonates are bolder and less risk aversive—i.e., they maintain low heart rates during human handling and have longer latencies to leave once released (Amin et al. 2021).

A few weeks after birth, most fawns make the transition from a solitary life to a group-living one and they join the female herd with their mothers, gradually shifting from a milk-based diet to a fully independent herbivorous diet (Chapman and Chapman 1997). When they join the main herd, fawns are expected to trade-off their time budgets, as typical for herbivores, between anti-predator behavior, i.e., scanning the landscape for potential threats, and resource acquisition, i.e., foraging (Sih 1980; Lima 1987; Bachman 1993). Simulations have recently shown that scanning dictates the amount of resources acquired and not vice versa (Sirot et al. 2021). Since boldness has been associated with spending less time scanning in fallow deer (Bergvall et al. 2011), scanning behavior could act as a feature of personality that in turn dictates resource acquisition. By spending less time scanning, individuals may be able to increase resource acquisition. Shedding light on these relationships will therefore provide a mechanism in which personality relates to resource acquisition during the early stages of independence in juveniles.

Here we tested whether neonate personality of fallow deer fawns, recorded during their hider phase, is related to the time they spend scanning while living in a group, during their first 6 months of life. We first tested whether scanning times were repeatable between individuals. Our main hypothesis was that among-individual differences in neonate traits would be covary with among-individual differences in scanning time. Specifically, we predicted that animals who react more boldly at capture, i.e., lower heart rates and longer latencies, also behave more boldly while in the herd, i.e., spend less time scanning. We then tested whether time spent scanning is inversely related to foraging time, our proxy for resource acquisition. Although this relationship is fairly clear in adults across vertebrates (Caro 2005), juveniles have been shown to scan the environment less than adults in several bird and mammal species (see Caro 2005 for a review), and could therefore also differ in their time budget trade-offs. We predicted that the trade-off between scanning and foraging would be present in fawns, and furthermore, that it would increase in strength when fawns grow older as they switch from a maternally provisioned nutrition rich (i.e., milk) to a nutritionally poorer (i.e., grazer) diet (Arenz and Leger 2000).

## METHODS

### Study site and study population

This study was conducted on a population of European fallow deer resident in the Phoenix Park, a 7 km^2^ enclosed park located near the center of Dublin, Ireland (53.3559° N, 6.3298° W). Vegetation in the park is predominately open grassland (~ 80%) with the remaining area composed by mixed woodland. Our study population of deer was estimated to be over 600 individuals over the course of this study (late summer estimates after the fawning). The majority of fawns are born from early June to early July. Fallow deer are a hider species and fawns remain hidden, usually in tall grass or understory vegetation, away from the main doe herd during the first 2–3 weeks of life following which they are brought into the doe herd by their mothers (Chapman and Chapman 1997; Ciuti et al. 2006). The only natural predator present in the park is the red fox (*Vulpes vulpes*), although fawns are also occasionally preyed upon by unleashed domestic dogs (*Canis lupus familiaris*). Deer are culled annually by professional stalkers over the winter period as part of the population management led by the Office of Public Works.

### Neonate captures

Fawns have routinely been captured and ear-tagged with unique numbered and colored plastic tags (Allflex medium, Mullinahone Co-op, Ireland) since the early 1970s as part of the monitoring and management of the herd (Hayden et al. 1992). Fawns were located by patrolling geographical areas traditionally used by does as fawning sites daily in June, when the majority of the births happen. Using fishing nets (1–1.5 m diameter; various brands), we located and tagged a total of 185 fawns over two consecutive years (*n* = 102 in 2018, *n* = 83 in 2019), of which 91 were recaptured once (*n* = 43 in 2018, *n* = 48 in 2019), 33 twice (*n* = 14 in 2018, *n* = 19 in 2019), and 9 three times or more (*n* = 4 in 2018, *n* = 5 in 2019). We recorded the following confounding variables which have been shown to affect neonatal response to handling (Amin et al. 2021): weight (in kg) was measured using a digital scale by laying the fawn in a 100-L bag (resolution: 0.01 kg—Dario Markenartikelvertrieb, Hamburg, Germany); air temperature was measured at the bed-site location using a digital thermometer (Grandbeing, China). We quantified the behavior of the fawn prior to capture (prior behavior) by recording whether the fawn was in motion (yes = 1, no = 0), turned its head to look around (yes = 1, no = 0), kept its head up or down (up = 1, down = 0), had its ears up or down (up = 1, down = 0), was down but got up (yes = 1, no = 0), and attempted to run away (yes = 1, no = 0). We took the mean of all these scores as a measure of prior behavior, where 1 indicated the most active behavior and 0 the least active behavior (*sensu*Amin et al. 2021).

Directly relevant to this study, we selected a physiological trait (heart rates prior to release, i.e., a physiological response of fawns to human handling) and a behavioral trait (latency to leave upon release), both shown to be repeatable at the among-individual level previously (Amin et al. 2021). Heart rates were taken directly before the weighting of the fawns and quantified by counting the number of beats per 20 s using a Lightweight Dual Head Stethoscope (MDF^®^, California, USA). The latency to leave (in s) on release was defined as the time it took the fawn to stand up after opening the weighing bag. We took 10 s as the maximum value and assigned that to individuals that had not moved before then (Amin et al. 2021). See Amin et al. (2021) for more details on the measure and variation of the neonate traits, and how these were affected by the environmental and conditional variables mentioned above.

### Focal observation in the herd

**Figure 1:**
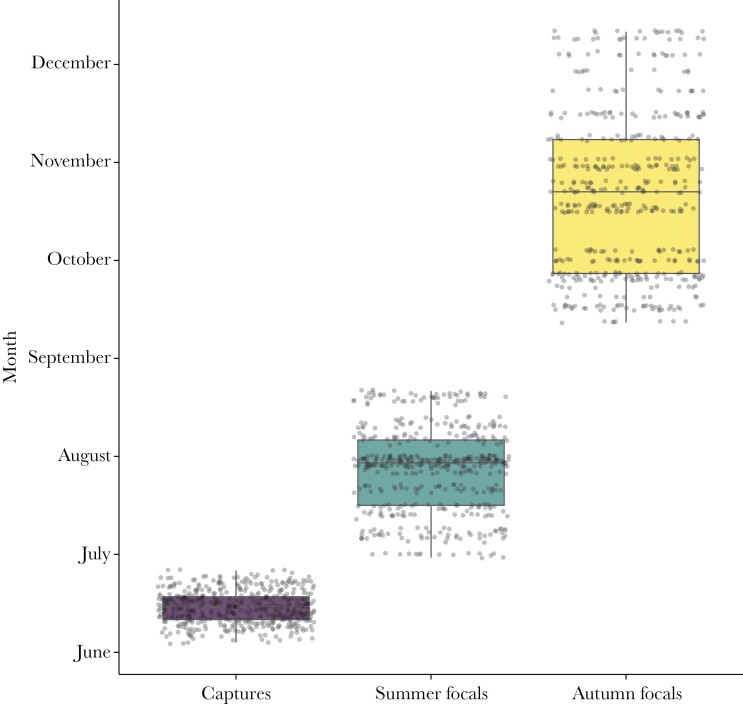
A temporal overview of the different data collection periods in 2018 and 2019, which were the neonate captures and the focal observations taken in summer and autumn. Jittered points indicate individual observations.

Time budgets were computed from focal sampling during summer and autumn in each year. Summer data collection took place in July and August of each year when newborn fawns join the female herd for the first time. Although the timing of emergence into the herd can be variable between individuals, most fawns make their first appearances in the herd in the summer months (see Supplementary S1). Autumn data collection took place from mid-September until early December, overlapping with the rutting season. The temporal overview of the different data collection periods is displayed in Fig.1.

Observations were taken between 09.00 and 17.00 h, generally in dry weather with high visibility. Focal subjects were observed using a spotting scope, at a distance no closer than 50 m, allowing the observer to maintain their distance and minimize their impact on the fawns’ behavior. Sampling of the focal individuals was random, with a priori determined rotation system used to find and sample most fawns available in the herd. We walked through the female day range and identified and selected a social group with active, non-resting fawns. A group was defined as multiple clustered individuals that were within 50 m of each other (Kasozi and Montgomery 2020). If a group included multiple active fawns, we selected the focal individual randomly. At the start of the observation, we recorded the total number of deer in that group. Groups were loosely aggregated, with constant fission-fusion throughout the day. The fawn was continuously observed for up to 25 min. Focals were often ended early due to fawns moving out of sight, i.e., laying down in the long grass, entering a traveling bout with the group, or a major herd disturbance. The fawn’s behavior during this period was recorded on a Dictaphone (Olympus VN-540PC) and transcribed later. The mean observation duration, excluding time out of sight, was 3.74 (SD = 3.98) min for the summer and 7.66 (SD = 5.81) min for the autumn season.

For each focal observation, we recorded the fawn’s position in the group at the start and end, based on the number of deer between the fawn and the edge of the group. This ranged from 0 to 3, with 0 being the fawn at the outermost edge of the herd, and 3 being three or more deer between the fawn and edge of the herd. We did so in order to account for potentially increased scanning rates of individuals observed at the edge of a group (Caro 2005). As a measure of human disturbance—and its potential effect on scanning rates (Ciuti et al. 2012), we also recorded the total amount of park visitors within a 50 m radius of the focal fawn during the observation. Once all active fawns in a herd had been recorded, the observers moved on, and another herd was selected. We avoided resampling the same fawns during the same session, unless the first observation was very short (< 2 min). Initially, fawns were chosen at random. As more focals were obtained, fawns were chosen more selectively, prioritizing individuals with a lesser number of observations. In total, we collected 477 focal observations on 137 fawns during summer and 430 focal observations on 141 fawns during autumn, making up a total of 907 focal observations with a total cumulative duration of 84.6 h.

Time budgets were extracted from the audio recordings using Jwatcher (Version 1.0) software. Twenty-six behaviors were recorded, a full description of each behavior can be found in Supplementary Table S1. Proportion of time spent on each behavior was calculated from the total time of each observation, excluding time spent out of sight. Proportion of time spent scanning, defined as standing still with the head above the shoulder height, was used as a measure of scanning time. We accumulated the time spent scanning while chewing and without chewing, since it was difficult to distinguish the two behaviors in the field. Proportion of time spent foraging was calculated by combining the proportions of time spent grazing, defined as unselectively feeding on grass and ground vegetation with the head below the shoulders, and browsing, defined as selectively feeding on leaves, bark, and top of plants (see Supplementary ST1 for full definitions).

### Ethical note

Captures and handling were carried out giving the highest priority to animal welfare. Fawns that were evidently newborn (a fully wet coat) were not captured and in such instances, we abandoned searches in that area to avoid disturbing the fawn. Gloves were always worn during handling to prevent transfer of human odors to the fawn (Galli et al. 2008). We operated in silence during animal handling and left the bed-site immediately after the release of the fawn. Fawns were released in a location adjacent to the capture site and facing in a direction away from the capture team. The capture, handling, tagging, and sampling of fawns was supervised by a certified and experienced wildlife biologist. Regular monitoring of the tagging regime has shown there are no survival implications in this population (see also Hayden et al. 1992). The focal data collection was observational: observers kept a minimum distance of 50m from the deer to avoid disturbing their behavior. The study protocol and all research procedures were approved by the Animal Research Ethics Committee (University College Dublin) under permit number AREC-E-18-28. All methods were in accordance with the Guidelines for the treatment of animals in behavioral research and teaching (Animal Behaviour 2020).

### Statistical analysis

All analyses were conducted using R 3.5.1 (R Core Team, 2020). To give a general overview of the analyses expanded upon below, repeatability (i.e., the fraction of total variance attributable to variation among individuals; Lessells and Boag 1987) of scanning and the covariance between neonate traits and time spent scanning were examined using bivariate mixed-effect models (Houslay and Wilson 2017). We then analyzed the trade-off between scanning and foraging using univariate mixed-effect models. All response variables and numerical explanatory variables were scaled prior to analysis, such that each variable was centered at their mean value and standardized to units of 1 phenotypic standard deviation. This has been recommended to improve model convergence and result interpretation (Houslay and Wilson 2017). Full details of the statistical analysis are provided below in the subsections.

### Neonate traits at capture and scanning time

To estimate the repeatability of and the among-individual covariation between the neonate traits at capture (heart rate and latency to leave) and scanning time while in the herd (in summer and autumn separately), we used multivariate mixed models, under a Bayesian MCMC framework, which are regarded as the state-of-the-art method for personality research (Houslay and Wilson 2017; Dingemanse and Wright 2020; Hertel et al. 2020). Multivariate mixed models were fitted via the *MCMCglmm-*package (Hadfield 2010). To determine whether either heart rate or latency to leave at capture were correlated with scanning behavior, we fitted four separate bivariate mixed models. Two of the models had heart rate and scanning time as response variables, one model for the summer period and the other for the autumn period. The other two models had latency to leave and scanning time as response variables, with also one model for the summer period and one model for the autumn period. Within-individual covariation between the two responses of each bivariate model were set to 0, since the two responses within each model were not measured at the same time (see Hadfield 2010). Correlation coefficients at the among-individual level (*r*_i_) and repeatability estimates, along with their 95% credible intervals, were computed following Houslay and Wilson (2017). In all models, Fawn ID was included as random intercept. For each bivariate model, we included only individuals that had at least one datapoint per response variable. We also omitted rows with missing values in any of the explanatory variables from the analysis.

In all cases we used a weakly informative prior [*R* = list(*V* = diag(2), nu = 0.002; *G* = list(*G*1 = list(*V* = diag(2), nu = 1.002))]. The neonate response variables (heart rate and latency to leave) were log-transformed prior to analysis to improve model fit and meet model assumptions regarding the gaussian distribution of errors. The scanning time response variable was in all cases logit-transformed, i.e., log(*y*/[1 − *y*]), as suggested by Warton and Hui (2011). Since the logit of 0 and 1 translate to ‐∞ and ∞, we added the smallest non-zero value to both the numerator and denominator of the logit equation (Warton and Hui 2011). We used a priori model structures for each response variable, which in the case of the neonate capture traits were based on a previous study (Amin et al. 2021). In the case of scanning time, we included explanatory variables that contained information on the context of each observation, where we included both the linear and quadratic terms for all the numerical explanatory variables to allow non-linear effects. To avoid over-fitting of the model, we simplified the full model by only removing the quadratic term of a variable when pMCMC > 0.1. The final model structures for each model are given in Tables 1 and 2, where the columns indicate the response variables and the rows the explanatory variables.

**Table 1:** Structure and output of the final bivariate models (MCMCglmm) used for the analysis of the among-individual covariation between heart rate at capture and time spent scanning in the summer (Heart rate-scanning summer model) as well the covariation between latency to leave at capture and time spent scanning in the summer (Latency-scanning summer model)

	Posterior mean [95% CrI]			
	Heart rate-scanning summer model		Latency-scanning summer model	
Variable	Heart rate	Scanning (summer)	Latency	Scanning (summer)
Intercept	‐0.162 [‐0.364, 0.044]	0.037 [‐0.130, 0.219]	‐0.643 [‐1.272, ‐0.068]	0.032 [‐0.143, 0.212]
Prior behavior	0.086 [0.009, 0.162]		‐0.128 [‐0.206, ‐0.049]	
Prior behavior^2^	‐0.070 [‐0.159, 0.007]			
Weight	0.219 [0.141, 0.295]		‐0.143 [‐0.223, ‐0.058]	
Weight^2^	0.032 [‐0.040, 0.108]		0.064 [‐0.001, 0.138]	
Year (2019)			0.103[‐0.146, 0.367]	
Capture			‐0.170 [‐0.322, ‐0.022]	
Time of day	0.127 [‐0.004, 0.259]	‐0.158 [‐0.256, ‐0.068]		‐0.160 [‐0.254, ‐0.064]
Time of day^2^	0.002 [‐0.108,0.105]			
Air temperature	0.079 [0.002, 0.152]			
Sex (m)	0.235 [‐0.023, 0.510]	‐0.315 [‐0.528, ‐0.093]		‐0.309 [‐0.517, ‐0.094]
Season (2018)		‐0.425 [‐0.649, ‐0.202]		‐0.417 [‐0.651, ‐0.188]
Number of people		0.073 [‐0.001, 0.149]		0.073 [‐0.002, 0.147]
Group size		0.089 [0.011, 0.164]		0.090 [0.010, 0.172]
Birthday (in days)		0.029 [‐0.058, 0.117]		0.025 [‐0.068, 0.114]
Days since emergence		‐0.264 [‐0.399, ‐0.120]		‐0.263 [‐0.402, ‐0.137]
Observation length (ms)		0.161 [0.080, 0.249]		0.159 [0.069, 0.241]
Observation length (ms)^2^		‐0.173 [‐0.250, ‐0.092]		‐0.174 [‐0.254, ‐0.091]

Posterior means with their associated 95% credible intervals of each of the explanatory variables (rows) included are given. Empty cells indicate that the explanatory variable was not included in the model for the respective response variables (model structures for the neonate traits defined by Amin et al. 2021). The position in the herd was not taken during the summer of 2018 and therefore, left out of the two models that were used for the summer season.

**Table 2:** Structure and output of the final bivariate models (MCMCglmm) used for the analysis of the among-individual covariation between heart rate at capture and time spent scanning in the autumn (Heart rate-scanning autumn model) as well the covariation between latency to leave at capture and time spent scanning in the autumn (Latency-scanning autumn model)

	Posterior mean [95% CrI]			
	Heart rate-scanning autumn model		Latency-scanning autumn model	
Variable	Heart rate	Scanning (autumn)	Latency	Scanning (autumn)
Intercept	‐0.147 [‐0.352, 0.062]	‐0.040 [‐0.248, 0.188]	‐0.977 [‐1.592, ‐0.415]	‐0.034 [‐0.254, 0.181]
Prior behavior	0.098 [0.018, 0.176]		‐0.133 [‐0.217, ‐0.054]	
Prior behavior^2^	‐0.028 [‐0.110, 0.059]			
Weight	0.188 [0.109, 0.274]		‐0.083 [‐0.167, ‐0.011]	
Weight^2^	0.025 [‐0.050, 0.103]		0.063 [‐0.011, 0.142]	
Year (2019)			0.132 [‐0.121, 0.410]	
Capture			‐0.252 [‐0.401, ‐0.102]	
Time of day	0.119 [‐0.007, 0.239]	‐0.149 [‐0.248, ‐0.042]		‐0.144 [‐0.252, ‐0.046]
Time of day^2^	0.010 [‐0.107, 0.134]			
Air temperature	0.081 [0.005, 0.161]			
Sex (m)	0.189 [‐0.071, 0.468]	‐0.083 [‐0.310, 0.141]		‐0.092 [‐0.332, 0.124]
Season (2018)		0.309 [0.084, 0.577]		0.304 [0.054, 0.532]
Number of people		0.050 [‐0.025, 0.121]		0.052 [‐0.020, 0.131]
Group size		0.051 [‐0.031, 0.129]		0.055 [‐0.027, 0.134]
Group size^2^		0.081 [0.006, 0.162]		0.074 [‐0.006, 0.151]
Birthday (in days)		‐0.004 [‐0.086, 0.085]		‐0.011 [‐0.093, 0.081]
Position in the herd		0.038 [‐0.042, 0.116]		0.037 [‐0.041, 0.123]
Position in the herd^2^		0.088 [0.006, 0.167]		0.089 [0.002, 0.175]
Days since emergence		‐0.238 [‐0.361, ‐0.109]		‐0.239 [‐0.366, ‐0.120]
Observation length (ms)		0.014 [‐0.075, 0.112]		0.020 [‐0.076, 0.109]

Posterior means with their associated 95% credible intervals of each of the explanatory variables (rows) included are given. Empty cells indicate that the explanatory variable was not included in the model for the respective response variables (model structure for neonate traits defined by Amin et al. 2021).

All MCMC-chains were run for a total length of 1,050,000 iterations, with a thinning of 500 and a burnin of the first 50,000 iterations, leading to a total of 2000 saved iterations. Model convergence was checked by running four separate chains for each bivariate model and calculating the multivariate scale reduction factor (Brooks and Gelman 1998), which never exceeded 1.1. We also visually inspected the chains, ensuring that every parameter had an effective sample size of at least 1000, and the autocorrelation of the posterior means and variances. From these, we concluded that the chains had converged properly and had negligible autocorrelations. Inferences concerning each of the correlations were made based on the posterior mean and the highest posterior density interval. We considered a relationship to be meaningful if less than 5% of the posterior distribution crossed zero (Allen et al. 2017; Jennings et al. 2018). To visualize the relationships between the responses of the bivariate, we extracted the posterior means of the random intercepts (BLUPs; Houslay and Wilson 2017). Full details on the bivariate models, including all the code, model summaries, and model diagnostics are given as supplementary material (Supplementary S2).

### Trade-off between scanning and foraging

To investigate the possible trade-off between scanning and foraging in young fawns and the possible change over ontogeny, we fitted a linear mixed-effect model (*lme4* package, Bates et al. 2015). Time spent scanning and time spent foraging were quantified as proportions of total time, which were then logit-transformed (Warton and Hui 2011). Since scanning is proposed to be driving resource acquisition (Sirot et al. 2021), we used foraging time as our response variable and scanning time as explanatory variable. To investigate change over time, we included the day of the year as a numerical explanatory variable, along with its interaction with scanning time. We included the quadratic terms of scanning time and day of the year to allow for non-linear effects. Finally, to correct for the effect of observation length on our estimates of foraging behavior (see sensitivity analysis below; Supplementary S3), we also included the duration of each observation as an explanatory variable. The predicted model effect following from this model was visualized using the *effects*-package with 95% marginal confidence intervals (Fox and Weisberg 2018, 2019).

### Sensitivity analysis

Initially, we aimed to include foraging time as a response variable in our bivariate models as well, in addition to scanning time and the neonate response variables, to investigate the relationship between neonate personality and resource acquisition directly. Prior to running our bivariate models, however, we investigated the stability of foraging time and scanning time estimates over different observation lengths. This was done because very short observations may produce biased time budgets (Childress and Lung 2003). For that purpose, we ran a sensitivity analysis (Supplementary S3). From the sensitivity analysis we concluded that foraging time was strongly affected by observation length and failed to stabilize even with increasing observation lengths. We therefore decided not to include foraging time as a response variable in our bivariate models, which we use to estimate among-individual covariation. Scanning time, on the other hand, was relatively robust and, especially in autumn, barely affected by observation length. There was some minor underestimation of scanning time for very short observations, mainly during summer, and we therefore included observation length as an explanatory variable in our bivariate models for scanning time.

## RESULTS

### Neonate traits at capture and scanning time

**Figure 2:**
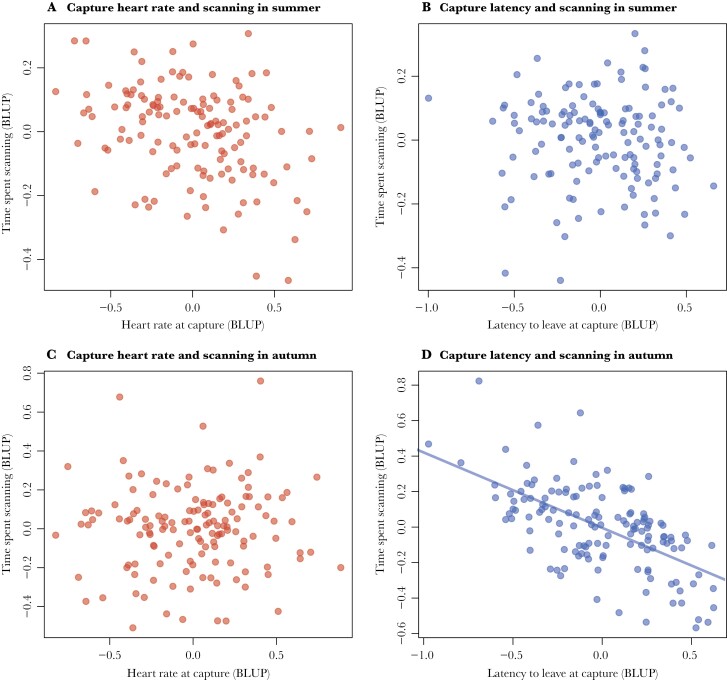
The relationships between (A) heart rate and scanning time in summer, (B) latency to leave and scanning time in summer, (C) heart rate and scanning time in autumn and (D) latency to leave and scanning time in autumn. Posterior means of the random intercepts (BLUPs) were used here for visualization purposes only. Solid trendline indicates a meaningful effect.

Both neonate traits measured at capture were found to be repeatable among individuals (heart rate: *R* = 0.35, 95% CrI [0.18, 0.52], *n* = 141 individual fawns; latency to leave: *R* = 0.33, 95% CrI [0.17, 0.48], *n* = 141). The proportion of time that fawns spent scanning was also repeatable among individuals, in both summer as well as autumn (summer: *R* = 0.12, 95% CrI [0.05, 0.18], *n* = 137; autumn: *R* = 0.17, 95% CrI [0.09, 0.25], *n* = 141). The posterior means and 95% CI of the explanatory variables used for estimating repeatability and among-individual covariance between neonate traits at capture and time spent scanning are given in Table 1 (summer models) and Table 2 (autumn models). We found no meaningful relationship between heart rates and scanning time in summer nor in autumn (Table 3; Figure 2A, C). There was also no clear pattern between latency to leave and scanning time in the summer (Table 3; Figure 2B). In autumn, however, we did find a meaningful negative relationship between latency to leave and scanning time. Individuals with higher latencies to leave as neonates in June spent less time scanning their environment in autumn (Table 3; Figure 2D).

**Table 3:** Correlations between different traits, at the among-individual level, extracted from bivariate models

Response 1	Response 2	Correlation coefficient (*r*_i_)	95% Credible intervals	*N*_fawns_
Heart rate	Scanning (summer)	‐0.163	[‐0.521, 0.225]	137
Latency	Scanning (summer)	‐0.035	[‐0.394, 0.374]	137
Heart rate	Scanning (autumn)	0.020	[‐0.344, 0.426]	141
Latency	Scanning (autumn)	‐**0.353**	**[**‐**0.674,** ‐**0.018]**	141

Correlations displayed in bold indicate statistically meaningful effects.

### Trade-off between scanning and foraging

**Figure 3:**
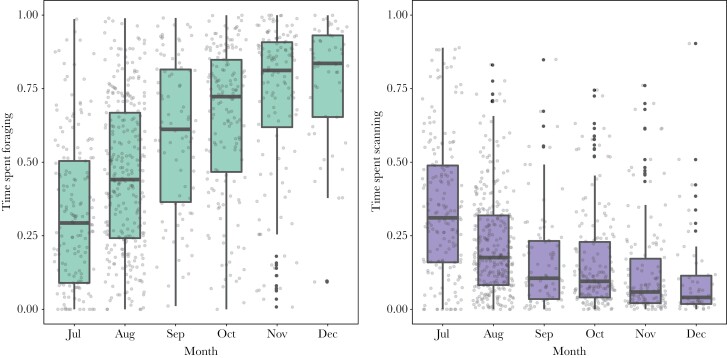
The increase in time spent foraging (left plot) and decrease in time spent scanning (right plot) of fallow deer fawns over the first 6 months of life. The times spent are given as proportions of total time of active bouts while in a group of deer.

**Figure 4:**
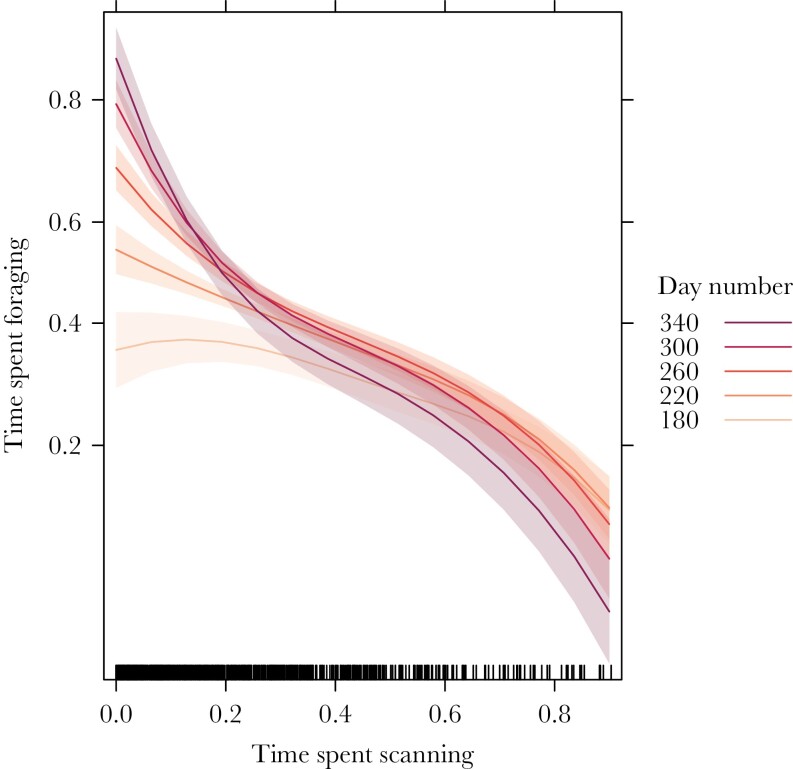
The relationship between the proportion of time spent scanning and the proportion of time spent foraging over time, i.e., when fawns gradually moved from a mainly milk-based diet to a grazer one (fully weaned). Predicted patterns (lines) surrounded by marginal 95% confidence intervals (shaded polygons) are derived from the linear mixed-effect model that was used for investigating the trade-off between scanning and foraging. This model included quadratic effects, which allow for effects that are not linear. Different time periods are indicated by different colors, with dates later in the year being represented by darker colors (day 180 = 29 June; day 220 = 8 August; day 260 = 17 September; day 300 = 27 October; day 340 = 6 December).

Fawns decreased their scanning time and increased their foraging time as they aged (see Figure 3), i.e., during the switch from a milk-based to a grazer diet (fully weaned). Suckling events per hour (s/h) were indeed high in summer (focal observations: 0.81 suckling/hour, range per month: 0.57–0.94 s/h) and nearly disappeared in autumn (focal observations: 0.15 s/h, range per month: 0.00–0.25 s/h). We investigated whether there was a trade-off between time spent scanning and time spent foraging and whether and how this developed over time. Time spent scanning negatively affected time spent foraging (linear term: *β* = ‐0.90 ± 0.06 SE, *P* < 0.001, *n* = 907 focal observations on *n* = 156 fawns; quadratic term: *β* = ‐0.47 ± 0.06 SE, *P* < 0.001, *n* = 907 focal observations on *n* = 156 fawns) and this association only became stronger over time (Figure 4), given the strong negative effect of the interaction between scanning time and days of the year (*β* = ‐0.20 ± 0.02 SE, *P* < 0.001, *n* = 907 focal observations on *n* = 156 fawns).

## DISCUSSION

Current debate within the field of animal personality focuses on the exact role that personality plays in maintaining life-history trade-offs (Laskowski et al. 2021; Haave-Audet et al. 2022). Contrary to what popular models such as POLS predict, recent meta-analyses have found no support for the prediction that bolder individuals survive less well (Royauté et al. 2018; Moiron et al. 2020; Haave-Audet et al. 2022). This suggests that bolder individuals may be able to acquire more resources (Laskowski et al. 2021), through which they can avoid paying a survival cost. Whether this relationship between personality and resource acquisition is present early in life or how it develops over ontogeny is, however, poorly understood. Here we shed light on this relationship and the development of among-individual differences in juveniles of a wild large mammal, by studying fallow deer fawns from birth until their sixth month of life. In line with our predictions, we found that repeatable among-individual differences in behavioral response of neonates were related to the time they spent scanning their environments while in the herd with their conspecifics, which also was repeatable among individuals. This scanning behavior was negatively related to time spent foraging, and this relationship only got stronger over time, suggesting among-individual differences in resource acquisition, through among-individual differences in time budget allocations. Contrary to our expectations, however, the relationship between neonate traits and time spent scanning was only present in autumn, but not earlier in summer, and also only involved the behavioral neonate response (i.e., latency to leave), and not the physiological response (i.e., heart rate). Altogether, our results show that among-individual differences are present shortly after birth and that these differences likely drive resource acquisition months later. This highlights a potential mechanistic pathway in which personality may lead to resource differences in the earliest stages of maturation.

Animal personality has been related to habitat use in other taxa. More explorative juvenile lemon sharks (*Negaprion brevirostris*) for instance, took more risks than less explorative individuals by swimming further from the shores in a subpopulation with low predator abundance (Dhellemmes et al. 2021). This enabled them to forage more efficiently, at the cost of higher exposure to predators. Similarly, bold golden-mantel ground squirrels (*Callospermophilus lateralis*) had larger core areas and occupied more perches in their areas than their shy counterparts (Aliperti et al. 2021). Bonnot et al. (2015) found that roe deer (*Capreolus capreolus*) that reacted less actively during capture and handling, also tended to use open habitats more than conspecifics that reacted more actively at capture. However, these studies mainly focused on habitat use, whereas we studied time investments regardless of habitat type or usage in a fairly homogenous environment. We show that time spent scanning was repeatable, with 12% (in summer) and 17% (in autumn) of the variation in scanning time being attributable to the among-individual level. This is possibly even more remarkable given the fact that fallow deer are social animals, where scanning behavior may be influenced by the behavior of conspecifics in the same group. Despite these group effects and other environmental factors, time investments differed consistently among individuals, indicating that certain individuals, namely those that spend less time scanning, systematically have more time to spend on foraging and subsequently, to gain more resources than other individuals.

The current scientific debate within the field of animal personality is focusing on whether among-individual differences in behavior mostly reflect among-individual differences in resource allocation or acquisition ( Haave-Audet et al. 2022; Laskowski et al. 2021). Recent meta-analyses seem to suggest the latter, since boldness was not found to be associated with a survival cost (Moiron et al. 2020; Haave-Audet et al. 2022). These analyses, however, do not investigate the direct relationship between personality and resource acquisition. In this study, we show how individual fawns with a longer latency to leave at capture also spent less time scanning their environments months later during autumn, while in the herd with adult deer. Both behaviors could be classified as bold: individuals that stay during a capture conserve energy at the cost of risking mortality; likewise, individuals that spend less time scanning in the herd have more time to gain resources at the cost of predator detection. Thus, our results show that personality differences, already present at the neonate stage, are maintained, and likely lead to systematic differences in resource acquisition. This can then lead to positive feedback loops, in which bold individuals can achieve and maintain higher state than their shy conspecifics (Luttbeg and Sih 2010; Sih et al. 2015), potentially allowing bolder individuals to reproduce and survive better overall (Van Noordwijk and De Jong 1986; Laskowski et al. 2021).

The relationship between neonate capture response and time spent scanning was, contrary to our expectations, not present earlier on during summer. During this summer period, fawns make their first entrances into the herd with adult deer, after spending the first weeks of life hiding in the vegetation (Chapman and Chapman 1997). In addition, fawns gradually switch from a nutrient rich diet (i.e., milk) to a nutrient poor diet (i.e., vegetation) with a concomitant need to invest more time in foraging. Fawns are thus very dependent on their mother for their resources during the first months and this dependency decreases with time, when their ability to forage successfully on their own becomes the main constraining factor for resource acquisition (Chapman and Chapman 1997). As a result, scanning behavior is expected to have a stronger limiting effect on foraging as fawns age, an effect clearly shown by our models. This suggests that scanning behavior may not be functionally linked to life-history differences (here: resource acquisition) in summer, when fawns are also more dependent on milk of their mother, whereas this relationship is present in autumn. Therefore, even though the same behavior was measured in summer and autumn, the functional role of that behavior could be very different between life-stages. This may explain why we found no clear relationship between neonate personality and scanning behavior in summer.

Another possibility is that relationships between different aspects of animal personality are overshadowed during major transitional phases in life. The emergence into the herd is such a major transition in the early-life of fawns, where they are suddenly in the presence of many other conspecifics. From that point onwards, fawns socialize with other deer, and will therefore be exposed to many new stimuli. Dairy cattle, for instance, showed long-term consistency before and after puberty, but not across (Neave et al. 2020). Similarly, among-individual differences in red junglefowl (*Gallus gallus*) chicks’ behavior were variable during ontogeny and stabilized after independence (Favati et al. 2016), a pattern also seen in wild fairy-wrens (*Malurus cyaneus*, Hall et al. 2015). On the other hand, there are also studies that do report long-term consistency across life-stages (Petelle et al. 2013; Debeffe et al. 2015). Petelle et al. (2013) show that yellow-bellied marmots (*Marmota flaviventris*) show long-term consistency in docility during captures, but not in boldness, whereas Debeffe et al. (2015) also show long-term consistency in docility, but then in wild roe deer (*Capreolus capreolus*) of which the youngest individuals were already months past their hiding phase. It is therefore possible that these studies found long-term consistency because they have not sampled individuals during transitional phases, but rather in between transitional phases. Even though heart rates and latency to leave are strongly and inversely correlated in neonates at captures (Amin et al. 2021), we found no pattern between heart rates at capture and time spent scanning, suggesting that these two metrics are measuring separate traits. Captures of wild animals can be a stressful event, and typically evoke an acute stress response in prey animals such as fallow deer, which leads to an increased physiological and behavioral response (Harris and Carr 2016). This relationship between physiology and behavior does not have to be present at other times, such as during foraging bouts where animals are expected to have lower anxiety levels, and therefore also lower HPA-axis activation (Harris and Carr 2016). Our findings in this study emphasize the need to include both physiological and behavioral responses to gain a better understanding of how physiology and behavior are (or are not) related in different contexts.

Adult herbivores are classically expected to trade-off their time investments between anti-predator behavior and resource acquisition. Although juveniles are not studied as extensively, previous research does indicate that juveniles differ from adults in the amount of time they spend scanning (Caro 2005). In most birds and mammals, juveniles are shown to spend less time scanning than adults (e.g., Alados 1985; Lashley et al. 2014; Li et al. 2015). The general explanation is that juveniles fail to recognize threats from predators and as a consequence spend less time scanning. In species where juveniles have a greater risk of being predated upon due to their reduced ability to escape, however, they may spend more time scanning due to the increased mortality threat (Caro 2005). Our results show that fallow deer juveniles follow this pattern: as fawns grew older, they reduced their time spent scanning. This decrease in scanning time was accompanied by an increase in time spent foraging, a natural consequence of the weaning process (Chapman and Chapman 1997).

To conclude, we have provided empirical support for the relationship between innate among-individual differences and resource acquisition, suggesting a mechanistic pathway in which personality is associated with life-history. We have done so in juveniles of a wild large mammal, which have received little attention in the literature compared to other taxa (Bell et al. 2009). We furthermore have highlighted the development of among-individual variation from birth, throughout the transition from a solitary lifestyle to a group-living one, up until the sixth month of life. Our results highlight how transitional phases can complicate patterns between behavior and life-history, thereby offering novel insights into the ontogeny of animal personality. Overall, our study emphasizes the importance of including ontogeny for future studies, and the necessity to understand the relationship between personality and acquisition for the improvement of theory in the field of animal personality.

## Supplementary Material

arac072_suppl_Supplementary_File_S1

arac072_suppl_Supplementary_File_S2

arac072_suppl_Supplementary_File_S3

arac072_suppl_Supplementary_File_ST1
