# Supplementary material for: Neonate personality affects early-life resource acquisition in a large social mammal
Source: Behav Ecol. 2022 Aug 9;33(5):1025–35. doi: 10.1093/beheco/arac072 (PMC9664924; doi:10.1093/beheco/arac072)
Supplement: arac072_suppl_Supplementary_File_S2 [file arac072_suppl_supplementary_file_s2.html]

S2: Full analysis of bivariate models reported in Amin et al.: Neonate personality affects early-life resource acquisition in a large social mammal


# S2: Full analysis of bivariate models reported in Amin et al.: Neonate personality affects early-life resource acquisition in a large social mammal

# Description:

This markdown contains the code to run the bivariate models as reported in the above paper, together with the full model outputs. The datasets that are used are created from the raw data (“Raw\_data.csv”), by use of the other script “SR1\_Create\_model\_datasets.R”.

This markdown is mainly divided into two sections: A) Creating final models and B) Results. Section A will use the datasets to create full models, then simplify them to the final models. Finally, diagnostics and model output will be checked. Section B will use the final models to reproduce the results reported in the main manuscript.

There is freedom to rerun models with the data and code provided here. In that case, we’d like to emphasize that the exact values of newly reran bivariate models may slightly differ from the values reported in the paper. This is entirely normal and due to the process of MCMC. However, all conclusions and main findings should be exaclty the same and should not be affected.

### Description of variable names used in this script and which variables they represent in the paper:

meancap = Prior behaviour as described in the manuscript X..Deer = Number of deer in the group X..People = Number of people within 50 meters (excluding observers) Capture = Capture number, i.e. whether it was the first, second, third, fourth or fifth capture of the same animal BDAY = The birthday of the fawn, as numerical variable (1-365) Days = Day of the year, as numerical variable (1-365)

# Section A: Creating final models

  We start the process of creating our models by loading the package and the datasets needed for the analysis. We correct the variable structures where needed after which the data is ready. We then create our weakly informative prior, after which everything is ready for our bivariate models. We will go through each model one by one, starting with the full model and ending with the final model.

```
library(MCMCglmm)


Latency_Summer = read.csv("Latency_Summer_final.csv")
Latency_Autumn = read.csv("Latency_Autumn_final.csv")
HR_Summer = read.csv("HR_Summer_final.csv")
HR_Autumn = read.csv("HR_Autumn_final.csv")

Latency_Summer$Variable = as.factor(Latency_Summer$Variable)
Latency_Summer$Year = as.factor(Latency_Summer$Year)
Latency_Summer$Season = as.factor(Latency_Summer$Season)
Latency_Summer$Sex = as.factor(Latency_Summer$Sex)
Latency_Summer$Variable = as.factor(ifelse(Latency_Summer$Variable == "Vigilance", "Scanning", as.character(Latency_Summer$Variable)))

Latency_Autumn$Variable = as.factor(Latency_Autumn$Variable)
Latency_Autumn$Year = as.factor(Latency_Autumn$Year)
Latency_Autumn$Season = as.factor(Latency_Autumn$Season)
Latency_Autumn$Sex = as.factor(Latency_Autumn$Sex)
Latency_Autumn$Variable = as.factor(ifelse(Latency_Autumn$Variable == "Vigilance", "Scanning", as.character(Latency_Autumn$Variable)))

HR_Summer$Variable = as.factor(HR_Summer$Variable)
HR_Summer$Year = as.factor(HR_Summer$Year)
HR_Summer$Season = as.factor(HR_Summer$Season)
HR_Summer$Sex = as.factor(HR_Summer$Sex)
HR_Summer$Variable = as.factor(ifelse(HR_Summer$Variable == "Vigilance", "Scanning", as.character(HR_Summer$Variable)))

HR_Autumn$Variable = as.factor(HR_Autumn$Variable)
HR_Autumn$Year = as.factor(HR_Autumn$Year)
HR_Autumn$Season = as.factor(HR_Autumn$Season)
HR_Autumn$Sex = as.factor(HR_Autumn$Sex)
HR_Autumn$Variable = as.factor(ifelse(HR_Autumn$Variable == "Vigilance", "Scanning", as.character(HR_Autumn$Variable)))

# Setting (uninformative) prior :

prior = list(R = list(V = diag(2), nu = 0.002), G = list(G1 = list(V = diag(2), nu = 1.002)))
```

### A.1) Heart rate-scanning summer model

We provide the code for the full model below.

```
HR_Summer_model<- MCMCglmm(Response~(Variable-1)+
                       at.level(Variable,1):scale(poly(meancap,2), scale = TRUE) +
                       at.level(Variable,1):scale(poly(Weight,2), scale = TRUE) +
                       at.level(Variable,1):scale(poly(Time,2), scale = TRUE) +
                       at.level(Variable,1):Sex +
                       at.level(Variable,1):scale(Air.temperature, scale = TRUE) +
                       at.level(Variable,2):Season +
                       at.level(Variable,2):scale(poly(Time,2), scale = TRUE) +
                       at.level(Variable,2):scale(poly(X..People,2), scale = TRUE) +
                       at.level(Variable,2):scale(poly(X..Deer, 2), scale = TRUE) + 
                       at.level(Variable,2):scale(poly(BDAY,2), scale = TRUE) +
                       at.level(Variable,2):scale(poly(Days_in_herd,2), scale = TRUE) +
                       at.level(Variable,2):scale(poly(Duration,2), scale = TRUE) +
                       at.level(Variable,2):Sex, 
                     random=~us(Variable):FawnID, rcov=~idh(Variable):units, family= "gaussian", prior=prior,nitt=1050000,thin=500,burnin=50000, data=HR_Summer,verbose=TRUE, pr = TRUE)
```

We have saved the model chain we used. We will load that model now and inspect the output.

```
HR_Summer_model <- readRDS ("HR_Summer_model.rds")
summary(HR_Summer_model)
```

```
## 
##  Iterations = 50001:1049501
##  Thinning interval  = 500
##  Sample size  = 2000 
## 
##  DIC: 1874.818 
## 
##  G-structure:  ~us(Variable):FawnID
## 
##                                          post.mean l-95% CI u-95% CI eff.samp
## VariableHRend:VariableHRend.FawnID         0.29179   0.1413  0.47205     2493
## VariableScanning:VariableHRend.FawnID     -0.02799  -0.0968  0.04183     2000
## VariableHRend:VariableScanning.FawnID     -0.02799  -0.0968  0.04183     2000
## VariableScanning:VariableScanning.FawnID   0.10787   0.0512  0.17029     2226
## 
##  R-structure:  ~idh(Variable):units
## 
##                        post.mean l-95% CI u-95% CI eff.samp
## VariableHRend.units       0.5100   0.3741   0.6562     2000
## VariableScanning.units    0.8195   0.7096   0.9360     2000
## 
##  Location effects: Response ~ (Variable - 1) + at.level(Variable, 1):scale(poly(meancap, 2), scale = TRUE) + at.level(Variable, 1):scale(poly(Weight, 2), scale = TRUE) + at.level(Variable, 1):scale(poly(Time, 2), scale = TRUE) + at.level(Variable, 1):Sex + at.level(Variable, 1):scale(Air.temperature, scale = TRUE) + at.level(Variable, 2):Season + at.level(Variable, 2):scale(poly(Time, 2), scale = TRUE) + at.level(Variable, 2):scale(poly(X..People, 2), scale = TRUE) + at.level(Variable, 2):scale(poly(X..Deer, 2), scale = TRUE) + 
##     at.level(Variable, 2):scale(poly(BDAY, 2), scale = TRUE) + at.level(Variable, 2):scale(poly(Days_in_herd, 2), scale = TRUE) + at.level(Variable, 2):scale(poly(Duration, 2), scale = TRUE) + at.level(Variable, 2):Sex 
## 
##                                                                    post.mean   l-95% CI   u-95% CI eff.samp  pMCMC    
## VariableHRend                                                     -1.647e-01 -3.715e-01  4.189e-02     2000  0.129    
## VariableScanning                                                   6.759e-03 -2.240e-01  2.019e-01     2286  0.955    
## at.level(Variable, 1):scale(poly(meancap, 2), scale = TRUE)1       8.557e-02  1.111e-02  1.611e-01     2000  0.028 *  
## at.level(Variable, 1):scale(poly(meancap, 2), scale = TRUE)2      -6.897e-02 -1.534e-01  1.140e-02     2174  0.104    
## at.level(Variable, 1):scale(poly(Weight, 2), scale = TRUE)1        2.200e-01  1.417e-01  2.999e-01     2000 <5e-04 ***
## at.level(Variable, 1):scale(poly(Weight, 2), scale = TRUE)2        3.449e-02 -3.738e-02  1.133e-01     2000  0.365    
## at.level(Variable, 1):scale(poly(Time, 2), scale = TRUE)1          1.277e-01  1.115e-03  2.470e-01     2172  0.041 *  
## at.level(Variable, 1):scale(poly(Time, 2), scale = TRUE)2         -3.764e-05 -1.103e-01  9.909e-02     2000  0.984    
## at.level(Variable, 1):Sexm                                         2.379e-01 -3.176e-02  5.046e-01     2000  0.091 .  
## at.level(Variable, 1):scale(Air.temperature, scale = TRUE)         7.874e-02 -7.793e-03  1.461e-01     1952  0.057 .  
## at.level(Variable, 2):SeasonSummer2018                            -4.749e-01 -7.317e-01 -2.255e-01     2000 <5e-04 ***
## scale(poly(Time, 2), scale = TRUE)1:at.level(Variable, 2)         -1.667e-01 -2.621e-01 -6.646e-02     2000  0.002 ** 
## scale(poly(Time, 2), scale = TRUE)2:at.level(Variable, 2)          4.585e-02 -5.264e-02  1.530e-01     2000  0.382    
## at.level(Variable, 2):scale(poly(X..People, 2), scale = TRUE)1     7.870e-02  2.484e-03  1.632e-01     2000  0.057 .  
## at.level(Variable, 2):scale(poly(X..People, 2), scale = TRUE)2     9.331e-03 -6.292e-02  8.434e-02     2219  0.782    
## at.level(Variable, 2):scale(poly(X..Deer, 2), scale = TRUE)1       9.458e-02  1.178e-02  1.788e-01     2000  0.029 *  
## at.level(Variable, 2):scale(poly(X..Deer, 2), scale = TRUE)2      -1.644e-02 -9.894e-02  6.818e-02     1836  0.701    
## at.level(Variable, 2):scale(poly(BDAY, 2), scale = TRUE)1          2.796e-02 -5.769e-02  1.138e-01     2000  0.560    
## at.level(Variable, 2):scale(poly(BDAY, 2), scale = TRUE)2         -1.341e-02 -9.947e-02  8.181e-02     2172  0.786    
## at.level(Variable, 2):scale(poly(Days_in_herd, 2), scale = TRUE)1 -3.418e-01 -5.659e-01 -1.248e-01     1811  0.005 ** 
## at.level(Variable, 2):scale(poly(Days_in_herd, 2), scale = TRUE)2 -7.247e-02 -2.249e-01  9.837e-02     2000  0.406    
## at.level(Variable, 2):scale(poly(Duration, 2), scale = TRUE)1      1.597e-01  7.923e-02  2.513e-01     2163  0.002 ** 
## at.level(Variable, 2):scale(poly(Duration, 2), scale = TRUE)2     -1.710e-01 -2.505e-01 -9.018e-02     2000 <5e-04 ***
## Sexm:at.level(Variable, 2)                                        -2.914e-01 -5.117e-01 -7.777e-02     1838  0.013 *  
## ---
## Signif. codes:  0 '***' 0.001 '**' 0.01 '*' 0.05 '.' 0.1 ' ' 1
```

Based on this full model, we now create our simplified final models. We do so by removing the quadratic effects which have a p > 0.1. Those are “Days in herd”, “BDAY”, “X..Deer”, “X..People” & “Time”. This model is now as following:

```
R_HR_Summer_model<- MCMCglmm(Response~(Variable-1)+
                             at.level(Variable,1):scale(poly(meancap,2), scale = TRUE) +
                             at.level(Variable,1):scale(poly(Weight,2), scale = TRUE) +
                             at.level(Variable,1):scale(poly(Time,2), scale = TRUE) +
                             at.level(Variable,1):Sex +
                             at.level(Variable,1):scale(Air.temperature, scale = TRUE) +
                             at.level(Variable,2):Season +
                             at.level(Variable,2):scale(Time, scale = TRUE) +
                             at.level(Variable,2):scale(X..People, scale = TRUE) +
                             at.level(Variable,2):scale(X..Deer, scale = TRUE) + 
                             at.level(Variable,2):scale(BDAY, scale = TRUE) +
                             at.level(Variable,2):scale(Days_in_herd, scale = TRUE) +
                             at.level(Variable,2):scale(poly(Duration,2), scale = TRUE) +
                             at.level(Variable,2):Sex, 
                           random=~us(Variable):FawnID, rcov=~idh(Variable):units, family= "gaussian", prior=prior,nitt=1050000,thin=500,burnin=50000, data=HR_Summer,verbose=TRUE, pr = TRUE)
```

We now load our model chain that we used for the main results in the manuscript, after which we give a model summary.

```
R_HR_Summer_model <- readRDS ("R_HR_Summer_model.rds")
summary(R_HR_Summer_model)
```

```
## 
##  Iterations = 50001:1049501
##  Thinning interval  = 500
##  Sample size  = 2000 
## 
##  DIC: 1866.903 
## 
##  G-structure:  ~us(Variable):FawnID
## 
##                                          post.mean l-95% CI u-95% CI eff.samp
## VariableHRend:VariableHRend.FawnID         0.29085  0.14995  0.45660     2000
## VariableScanning:VariableHRend.FawnID     -0.02939 -0.09970  0.04035     1887
## VariableHRend:VariableScanning.FawnID     -0.02939 -0.09970  0.04035     1887
## VariableScanning:VariableScanning.FawnID   0.10841  0.04902  0.17002     2000
## 
##  R-structure:  ~idh(Variable):units
## 
##                        post.mean l-95% CI u-95% CI eff.samp
## VariableHRend.units       0.5101   0.3817   0.6567     2429
## VariableScanning.units    0.8137   0.7015   0.9242     2000
## 
##  Location effects: Response ~ (Variable - 1) + at.level(Variable, 1):scale(poly(meancap, 2), scale = TRUE) + at.level(Variable, 1):scale(poly(Weight, 2), scale = TRUE) + at.level(Variable, 1):scale(poly(Time, 2), scale = TRUE) + at.level(Variable, 1):Sex + at.level(Variable, 1):scale(Air.temperature, scale = TRUE) + at.level(Variable, 2):Season + at.level(Variable, 2):scale(Time, scale = TRUE) + at.level(Variable, 2):scale(X..People, scale = TRUE) + at.level(Variable, 2):scale(X..Deer, scale = TRUE) + at.level(Variable, 
##     2):scale(BDAY, scale = TRUE) + at.level(Variable, 2):scale(Days_in_herd, scale = TRUE) + at.level(Variable, 2):scale(poly(Duration, 2), scale = TRUE) + at.level(Variable, 2):Sex 
## 
##                                                               post.mean  l-95% CI  u-95% CI eff.samp  pMCMC    
## VariableHRend                                                 -0.161546 -0.364350  0.044369     2000  0.127    
## VariableScanning                                               0.036766 -0.130180  0.219138     2329  0.696    
## at.level(Variable, 1):scale(poly(meancap, 2), scale = TRUE)1   0.085735  0.008906  0.161953     2257  0.031 *  
## at.level(Variable, 1):scale(poly(meancap, 2), scale = TRUE)2  -0.069958 -0.159072  0.007374     2000  0.095 .  
## at.level(Variable, 1):scale(poly(Weight, 2), scale = TRUE)1    0.219138  0.140691  0.295308     2000 <5e-04 ***
## at.level(Variable, 1):scale(poly(Weight, 2), scale = TRUE)2    0.032102 -0.040416  0.107925     2000  0.404    
## at.level(Variable, 1):scale(poly(Time, 2), scale = TRUE)1      0.126528  0.003516  0.258785     1845  0.049 *  
## at.level(Variable, 1):scale(poly(Time, 2), scale = TRUE)2      0.001708 -0.108136  0.105257     2000  0.958    
## at.level(Variable, 1):Sexm                                     0.234865 -0.023225  0.510168     2156  0.097 .  
## at.level(Variable, 1):scale(Air.temperature, scale = TRUE)     0.078637  0.001590  0.151554     2000  0.041 *  
## at.level(Variable, 2):SeasonSummer2018                        -0.425226 -0.649441 -0.202054     2000 <5e-04 ***
## at.level(Variable, 2):scale(Time, scale = TRUE)               -0.158015 -0.256046 -0.067996     2000  0.001 ***
## at.level(Variable, 2):scale(X..People, scale = TRUE)           0.073192 -0.001414  0.149123     2000  0.054 .  
## at.level(Variable, 2):scale(X..Deer, scale = TRUE)             0.089311  0.011364  0.164037     2000  0.025 *  
## at.level(Variable, 2):scale(BDAY, scale = TRUE)                0.029488 -0.058227  0.116903     1645  0.502    
## at.level(Variable, 2):scale(Days_in_herd, scale = TRUE)       -0.263583 -0.399110 -0.120167     2000 <5e-04 ***
## at.level(Variable, 2):scale(poly(Duration, 2), scale = TRUE)1  0.160849  0.079896  0.249072     2193 <5e-04 ***
## at.level(Variable, 2):scale(poly(Duration, 2), scale = TRUE)2 -0.173000 -0.249653 -0.091746     2000 <5e-04 ***
## Sexm:at.level(Variable, 2)                                    -0.315241 -0.527967 -0.093298     2136  0.003 ** 
## ---
## Signif. codes:  0 '***' 0.001 '**' 0.01 '*' 0.05 '.' 0.1 ' ' 1
```

We then check for chain convergence. We have ran separate chains which we load here, but feel free to run new chains of your own. instead.

```
mean(summary(R_HR_Summer_model)$Gcovariances[,4])
```

```
## [1] 1943.559
```

```
mean(abs(autocorr.diag(R_HR_Summer_model$VCV[,1:4], lag=c(1))))
```

```
## [1] 0.01209108
```

```
R_HR_Summer_model2 <- readRDS ("R_HR_Summer_model2.rds")
R_HR_Summer_model3 <- readRDS ("R_HR_Summer_model3.rds")
R_HR_Summer_model4 <- readRDS ("R_HR_Summer_model4.rds")

diag = gelman.diag(mcmc.list(R_HR_Summer_model$Sol, R_HR_Summer_model2$Sol, R_HR_Summer_model3$Sol, R_HR_Summer_model4$Sol ))
diag$mpsrf
```

```
## [1] 1.060869
```

Everything seems okay. We have finalized this model.

### A.2) Latency-scanning summer model

We provide the code for the full model below.

```
Lat_Summer<- MCMCglmm(Response~(Variable-1)+
                        at.level(Variable,1):scale(meancap, scale = TRUE) +
                        at.level(Variable,1):scale(poly(Weight,2), scale = TRUE) +
                        at.level(Variable,1):Year +
                        at.level(Variable,1):scale(Capture, scale = FALSE) +
                        at.level(Variable,2):Season +
                        at.level(Variable,2):scale(poly(Time,2), scale = TRUE) +
                        at.level(Variable,2):scale(poly(X..People,2), scale = TRUE) +
                        at.level(Variable,2):scale(poly(X..Deer, 2), scale = TRUE) + 
                        at.level(Variable,2):scale(poly(BDAY,2), scale = TRUE) +
                        at.level(Variable,2):scale(poly(Days_in_herd,2), scale = TRUE) +
                        at.level(Variable,2):scale(poly(Duration,2), scale = TRUE) +
                        at.level(Variable,2):Sex, 
                      random=~us(Variable):FawnID, rcov=~idh(Variable):units, family= "gaussian", prior=prior,nitt=1050000,thin=500,burnin=50000, data=Latency_Summer,verbose=TRUE, pr = TRUE)
```

We have saved the model chain we used. We will load that model now and inspect the output.

```
Lat_Summer <- readRDS ("Lat_Summer_model.rds")
summary(Lat_Summer)
```

```
## 
##  Iterations = 50001:1049501
##  Thinning interval  = 500
##  Sample size  = 2000 
## 
##  DIC: 1862.586 
## 
##  G-structure:  ~us(Variable):FawnID
## 
##                                          post.mean l-95% CI u-95% CI eff.samp
## VariableLatency:VariableLatency.FawnID    0.244244  0.12383  0.39009     2262
## VariableScanning:VariableLatency.FawnID  -0.005264 -0.06989  0.05823     2000
## VariableLatency:VariableScanning.FawnID  -0.005264 -0.06989  0.05823     2000
## VariableScanning:VariableScanning.FawnID  0.105137  0.05242  0.16894     1855
## 
##  R-structure:  ~idh(Variable):units
## 
##                        post.mean l-95% CI u-95% CI eff.samp
## VariableLatency.units     0.5055   0.3774   0.6392     2000
## VariableScanning.units    0.8197   0.7054   0.9316     2000
## 
##  Location effects: Response ~ (Variable - 1) + at.level(Variable, 1):scale(meancap, scale = TRUE) + at.level(Variable, 1):scale(poly(Weight, 2), scale = TRUE) + at.level(Variable, 1):Year + at.level(Variable, 1):scale(Capture, scale = FALSE) + at.level(Variable, 2):Season + at.level(Variable, 2):scale(poly(Time, 2), scale = TRUE) + at.level(Variable, 2):scale(poly(X..People, 2), scale = TRUE) + at.level(Variable, 2):scale(poly(X..Deer, 2), scale = TRUE) + at.level(Variable, 2):scale(poly(BDAY, 2), scale = TRUE) + at.level(Variable, 
##     2):scale(poly(Days_in_herd, 2), scale = TRUE) + at.level(Variable, 2):scale(poly(Duration, 2), scale = TRUE) + at.level(Variable, 2):Sex 
## 
##                                                                   post.mean  l-95% CI  u-95% CI eff.samp  pMCMC    
## VariableLatency                                                   -0.647809 -1.285473 -0.095706     2000  0.039 *  
## VariableScanning                                                   0.002466 -0.208403  0.221792     1975  0.985    
## at.level(Variable, 1):scale(meancap, scale = TRUE)                -0.130586 -0.200406 -0.053196     2000 <5e-04 ***
## at.level(Variable, 1):scale(poly(Weight, 2), scale = TRUE)1       -0.140625 -0.222815 -0.060268     1827  0.001 ***
## at.level(Variable, 1):scale(poly(Weight, 2), scale = TRUE)2        0.063473 -0.012328  0.130869     2000  0.081 .  
## at.level(Variable, 1):Year2019                                     0.097939 -0.164820  0.352052     1674  0.486    
## at.level(Variable, 1):scale(Capture, scale = FALSE)               -0.171833 -0.315822 -0.022972     2000  0.028 *  
## at.level(Variable, 2):SeasonSummer2018                            -0.467562 -0.704966 -0.209251     2000 <5e-04 ***
## at.level(Variable, 2):scale(poly(Time, 2), scale = TRUE)1         -0.175008 -0.278724 -0.080735     1850 <5e-04 ***
## at.level(Variable, 2):scale(poly(Time, 2), scale = TRUE)2          0.046550 -0.050365  0.148916     2000  0.385    
## at.level(Variable, 2):scale(poly(X..People, 2), scale = TRUE)1     0.079670  0.005987  0.164913     2000  0.048 *  
## at.level(Variable, 2):scale(poly(X..People, 2), scale = TRUE)2     0.010999 -0.063343  0.084773     2000  0.785    
## at.level(Variable, 2):scale(poly(X..Deer, 2), scale = TRUE)1       0.092533  0.012100  0.175267     2000  0.025 *  
## at.level(Variable, 2):scale(poly(X..Deer, 2), scale = TRUE)2      -0.015739 -0.104525  0.068211     2000  0.702    
## at.level(Variable, 2):scale(poly(BDAY, 2), scale = TRUE)1          0.024102 -0.065751  0.113835     2000  0.577    
## at.level(Variable, 2):scale(poly(BDAY, 2), scale = TRUE)2         -0.016363 -0.114494  0.080296     2000  0.733    
## at.level(Variable, 2):scale(poly(Days_in_herd, 2), scale = TRUE)1 -0.337868 -0.565225 -0.123250     2026  0.004 ** 
## at.level(Variable, 2):scale(poly(Days_in_herd, 2), scale = TRUE)2 -0.069211 -0.232123  0.086872     2000  0.405    
## at.level(Variable, 2):scale(poly(Duration, 2), scale = TRUE)1      0.161734  0.077593  0.254873     2000 <5e-04 ***
## at.level(Variable, 2):scale(poly(Duration, 2), scale = TRUE)2     -0.173208 -0.256066 -0.093578     2000 <5e-04 ***
## at.level(Variable, 2):Sexm                                        -0.286835 -0.494763 -0.052464     2000  0.012 *  
## ---
## Signif. codes:  0 '***' 0.001 '**' 0.01 '*' 0.05 '.' 0.1 ' ' 1
```

Based on this full model, we now create our simplified final models. We do so by removing the quadratic effects which have a p > 0.1. Those are “Days in herd”, “BDAY”, “X..Deer”, “X..People” & “Time”. This model is now as following:

```
R_Lat_Summer<- MCMCglmm(Response~(Variable-1)+
                        at.level(Variable,1):scale(meancap, scale = TRUE) +
                        at.level(Variable,1):scale(poly(Weight,2), scale = TRUE) +
                        at.level(Variable,1):Year +
                        at.level(Variable,1):scale(Capture, scale = FALSE) +
                        at.level(Variable,2):Season +
                        at.level(Variable,2):scale(Time, scale = TRUE) +
                        at.level(Variable,2):scale(X..People, scale = TRUE) +
                        at.level(Variable,2):scale(X..Deer, scale = TRUE) + 
                        at.level(Variable,2):scale(BDAY, scale = TRUE) +
                        at.level(Variable,2):scale(Days_in_herd, scale = TRUE) +
                        at.level(Variable,2):scale(poly(Duration,2), scale = TRUE) +
                        at.level(Variable,2):Sex, 
                      random=~us(Variable):FawnID, rcov=~idh(Variable):units, family= "gaussian", prior=prior,nitt=1050000,thin=500,burnin=50000, data=Latency_Summer,verbose=TRUE, pr = TRUE)
```

We now load our model chain that we used for the main results in the manuscript, after which we give a model summary.

```
R_Lat_Summer <- readRDS ("R_Lat_Summer_model.rds")
summary(R_Lat_Summer)
```

```
## 
##  Iterations = 50001:1049501
##  Thinning interval  = 500
##  Sample size  = 2000 
## 
##  DIC: 1854.849 
## 
##  G-structure:  ~us(Variable):FawnID
## 
##                                          post.mean l-95% CI u-95% CI eff.samp
## VariableLatency:VariableLatency.FawnID     0.24261   0.1186  0.38345     2000
## VariableScanning:VariableLatency.FawnID   -0.00517  -0.0707  0.05813     2997
## VariableLatency:VariableScanning.FawnID   -0.00517  -0.0707  0.05813     2997
## VariableScanning:VariableScanning.FawnID   0.10709   0.0557  0.17141     2000
## 
##  R-structure:  ~idh(Variable):units
## 
##                        post.mean l-95% CI u-95% CI eff.samp
## VariableLatency.units     0.5067   0.3911   0.6484     2000
## VariableScanning.units    0.8134   0.7109   0.9322     1979
## 
##  Location effects: Response ~ (Variable - 1) + at.level(Variable, 1):scale(meancap, scale = TRUE) + at.level(Variable, 1):scale(poly(Weight, 2), scale = TRUE) + at.level(Variable, 1):Year + at.level(Variable, 1):scale(Capture, scale = FALSE) + at.level(Variable, 2):Season + at.level(Variable, 2):scale(Time, scale = TRUE) + at.level(Variable, 2):scale(X..People, scale = TRUE) + at.level(Variable, 2):scale(X..Deer, scale = TRUE) + at.level(Variable, 2):scale(BDAY, scale = TRUE) + at.level(Variable, 2):scale(Days_in_herd, 
##     scale = TRUE) + at.level(Variable, 2):scale(poly(Duration, 2), scale = TRUE) + at.level(Variable, 2):Sex 
## 
##                                                               post.mean  l-95% CI  u-95% CI eff.samp  pMCMC    
## VariableLatency                                               -0.643403 -1.272470 -0.068411     2150  0.038 *  
## VariableScanning                                               0.031910 -0.143087  0.212196     2000  0.705    
## at.level(Variable, 1):scale(meancap, scale = TRUE)            -0.127542 -0.205850 -0.048954     2000  0.001 ***
## at.level(Variable, 1):scale(poly(Weight, 2), scale = TRUE)1   -0.143103 -0.222822 -0.058038     2000  0.002 ** 
## at.level(Variable, 1):scale(poly(Weight, 2), scale = TRUE)2    0.063859 -0.001092  0.137857     2857  0.076 .  
## at.level(Variable, 1):Year2019                                 0.103157 -0.146351  0.366990     2000  0.428    
## at.level(Variable, 1):scale(Capture, scale = FALSE)           -0.170303 -0.322410 -0.022126     2147  0.023 *  
## at.level(Variable, 2):SeasonSummer2018                        -0.417053 -0.651012 -0.188172     2000  0.001 ***
## at.level(Variable, 2):scale(Time, scale = TRUE)               -0.160041 -0.254406 -0.064390     2000 <5e-04 ***
## at.level(Variable, 2):scale(X..People, scale = TRUE)           0.073156 -0.001679  0.147015     2000  0.050 .  
## at.level(Variable, 2):scale(X..Deer, scale = TRUE)             0.090233  0.010346  0.171991     2000  0.037 *  
## at.level(Variable, 2):scale(BDAY, scale = TRUE)                0.025218 -0.068132  0.113615     2000  0.590    
## at.level(Variable, 2):scale(Days_in_herd, scale = TRUE)       -0.262975 -0.402114 -0.137322     2483  0.001 ***
## at.level(Variable, 2):scale(poly(Duration, 2), scale = TRUE)1  0.159347  0.069078  0.240709     1861 <5e-04 ***
## at.level(Variable, 2):scale(poly(Duration, 2), scale = TRUE)2 -0.174427 -0.253555 -0.091156     2000 <5e-04 ***
## at.level(Variable, 2):Sexm                                    -0.309232 -0.516684 -0.094059     2000  0.006 ** 
## ---
## Signif. codes:  0 '***' 0.001 '**' 0.01 '*' 0.05 '.' 0.1 ' ' 1
```

We then check for chain convergence. We have ran separate chains which we load here, but feel free to run new chains of your own. instead.

```
mean(summary(R_Lat_Summer)$Gcovariances[,4])
```

```
## [1] 2498.702
```

```
mean(abs(autocorr.diag(R_Lat_Summer$VCV[,1:4], lag=c(1))))
```

```
## [1] 0.01109342
```

```
R_Lat_Summer2 <- readRDS ("R_Lat_Summer_model2.rds")
R_Lat_Summer3 <- readRDS ("R_Lat_Summer_model3.rds")
R_Lat_Summer4 <- readRDS ("R_Lat_Summer_model4.rds")

diag = gelman.diag(mcmc.list(R_Lat_Summer$Sol, R_Lat_Summer2$Sol, R_Lat_Summer3$Sol, R_Lat_Summer4$Sol ))
diag$mpsrf
```

```
## [1] 1.050737
```

Everything seems okay. We have finalized this model.

### A.3) Heart rate-scanning autumn model

We provide the code for the full model below.

```
HR_Autumn_model<- MCMCglmm(Response~(Variable-1)+
                       at.level(Variable,1):scale(poly(meancap,2), scale = TRUE) +
                       at.level(Variable,1):scale(poly(Weight,2), scale = TRUE) +
                       at.level(Variable,1):scale(poly(Time,2), scale = TRUE) +
                       at.level(Variable,1):Sex +
                       at.level(Variable,1):scale(Air.temperature, scale = TRUE) +
                       at.level(Variable,2):Season +
                       at.level(Variable,2):scale(poly(Time,2), scale = TRUE) +
                       at.level(Variable,2):scale(poly(X..People,2), scale = TRUE) +
                       at.level(Variable,2):scale(poly(X..Deer, 2), scale = TRUE) + 
                       at.level(Variable,2):scale(poly(BDAY,2), scale = TRUE) +
                       at.level(Variable,2):scale(poly(Days_in_herd,2), scale = TRUE) +
                       at.level(Variable,2):scale(poly(Duration,2), scale = TRUE) +
                       at.level(Variable,2):Sex, 
                     random=~us(Variable):FawnID, rcov=~idh(Variable):units, family= "gaussian", prior=prior,nitt=1050000,thin=500,burnin=50000, data=HR_Autumn,verbose=TRUE, pr = TRUE)
```

We have saved the model chain we used. We will load that model now and inspect the output.

```
HR_Autumn_model <- readRDS ("HR_Autumn_model.rds")
summary(HR_Autumn_model)
```

```
## 
##  Iterations = 50001:1049501
##  Thinning interval  = 500
##  Sample size  = 2000 
## 
##  DIC: 1790.925 
## 
##  G-structure:  ~us(Variable):FawnID
## 
##                                          post.mean l-95% CI u-95% CI eff.samp
## VariableHRend:VariableHRend.FawnID        0.288212  0.13478  0.45704     2000
## VariableScanning:VariableHRend.FawnID     0.001642 -0.08966  0.07726     2000
## VariableHRend:VariableScanning.FawnID     0.001642 -0.08966  0.07726     2000
## VariableScanning:VariableScanning.FawnID  0.159378  0.07707  0.25361     2030
## 
##  R-structure:  ~idh(Variable):units
## 
##                        post.mean l-95% CI u-95% CI eff.samp
## VariableHRend.units       0.5215   0.3835   0.6700     2086
## VariableScanning.units    0.8035   0.6765   0.9262     2000
## 
##  Location effects: Response ~ (Variable - 1) + at.level(Variable, 1):scale(poly(meancap, 2), scale = TRUE) + at.level(Variable, 1):scale(poly(Weight, 2), scale = TRUE) + at.level(Variable, 1):scale(poly(Time, 2), scale = TRUE) + at.level(Variable, 1):Sex + at.level(Variable, 1):scale(Air.temperature, scale = TRUE) + at.level(Variable, 2):Season + at.level(Variable, 2):scale(poly(Time, 2), scale = TRUE) + at.level(Variable, 2):scale(poly(X..People, 2), scale = TRUE) + at.level(Variable, 2):scale(poly(X..Deer, 2), scale = TRUE) + 
##     at.level(Variable, 2):scale(poly(BDAY, 2), scale = TRUE) + at.level(Variable, 2):scale(poly(Position, 2), scale = TRUE) + at.level(Variable, 2):scale(poly(Days_in_herd, 2), scale = TRUE) + at.level(Variable, 2):scale(poly(Duration, 2), scale = TRUE) + at.level(Variable, 2):Sex 
## 
##                                                                    post.mean   l-95% CI   u-95% CI eff.samp  pMCMC    
## VariableHRend                                                     -1.478e-01 -3.398e-01  4.020e-02     2000  0.126    
## VariableScanning                                                  -3.605e-02 -2.558e-01  2.047e-01     2000  0.762    
## at.level(Variable, 1):scale(poly(meancap, 2), scale = TRUE)1       9.630e-02  1.183e-02  1.707e-01     1799  0.021 *  
## at.level(Variable, 1):scale(poly(meancap, 2), scale = TRUE)2      -2.726e-02 -1.108e-01  5.727e-02     1997  0.523    
## at.level(Variable, 1):scale(poly(Weight, 2), scale = TRUE)1        1.885e-01  1.043e-01  2.730e-01     2000 <5e-04 ***
## at.level(Variable, 1):scale(poly(Weight, 2), scale = TRUE)2        2.696e-02 -4.626e-02  1.067e-01     2155  0.474    
## at.level(Variable, 1):scale(poly(Time, 2), scale = TRUE)1          1.194e-01  2.144e-03  2.415e-01     2000  0.048 *  
## at.level(Variable, 1):scale(poly(Time, 2), scale = TRUE)2          1.146e-02 -1.087e-01  1.343e-01     1898  0.840    
## at.level(Variable, 1):Sexm                                         1.857e-01 -6.768e-02  4.490e-01     2140  0.170    
## at.level(Variable, 1):scale(Air.temperature, scale = TRUE)         8.368e-02  2.501e-03  1.621e-01     2000  0.041 *  
## at.level(Variable, 2):SeasonAutumnwinter2018                       2.756e-01  2.652e-02  5.365e-01     2000  0.030 *  
## scale(poly(Time, 2), scale = TRUE)1:at.level(Variable, 2)         -1.472e-01 -2.590e-01 -4.748e-02     2000  0.009 ** 
## scale(poly(Time, 2), scale = TRUE)2:at.level(Variable, 2)          5.194e-02 -2.865e-02  1.447e-01     2000  0.239    
## at.level(Variable, 2):scale(poly(X..People, 2), scale = TRUE)1     3.931e-02 -4.013e-02  1.137e-01     2000  0.306    
## at.level(Variable, 2):scale(poly(X..People, 2), scale = TRUE)2    -9.958e-03 -8.511e-02  6.492e-02     2170  0.781    
## at.level(Variable, 2):scale(poly(X..Deer, 2), scale = TRUE)1       4.264e-02 -4.030e-02  1.229e-01     2000  0.325    
## at.level(Variable, 2):scale(poly(X..Deer, 2), scale = TRUE)2       7.979e-02 -1.228e-02  1.581e-01     2000  0.065 .  
## at.level(Variable, 2):scale(poly(BDAY, 2), scale = TRUE)1         -7.608e-03 -9.920e-02  8.083e-02     2000  0.859    
## at.level(Variable, 2):scale(poly(BDAY, 2), scale = TRUE)2         -7.327e-02 -1.692e-01  2.748e-02     2000  0.154    
## at.level(Variable, 2):scale(poly(Position, 2), scale = TRUE)1      3.932e-02 -4.190e-02  1.202e-01     1842  0.332    
## at.level(Variable, 2):scale(poly(Position, 2), scale = TRUE)2      7.961e-02  7.102e-05  1.641e-01     2000  0.051 .  
## at.level(Variable, 2):scale(poly(Days_in_herd, 2), scale = TRUE)1 -2.515e-01 -3.818e-01 -1.114e-01     2000 <5e-04 ***
## at.level(Variable, 2):scale(poly(Days_in_herd, 2), scale = TRUE)2  4.018e-03 -1.361e-01  1.440e-01     1869  0.955    
## at.level(Variable, 2):scale(poly(Duration, 2), scale = TRUE)1      4.378e-02 -6.785e-02  1.505e-01     2000  0.443    
## at.level(Variable, 2):scale(poly(Duration, 2), scale = TRUE)2     -5.452e-02 -1.527e-01  4.543e-02     2000  0.289    
## Sexm:at.level(Variable, 2)                                        -5.366e-02 -3.041e-01  1.639e-01     2000  0.650    
## ---
## Signif. codes:  0 '***' 0.001 '**' 0.01 '*' 0.05 '.' 0.1 ' ' 1
```

Based on this full model, we now create our simplified final models. We do so by removing the quadratic effects which have a p > 0.1. Those are “Duration”, “Days in herd”, “BDAY”, “X..People”, “Time”. This model is now as following:

```
R_HR_Autumn_model<- MCMCglmm(Response~(Variable-1)+
                             at.level(Variable,1):scale(poly(meancap,2), scale = TRUE) +
                             at.level(Variable,1):scale(poly(Weight,2), scale = TRUE) +
                             at.level(Variable,1):scale(poly(Time,2), scale = TRUE) +
                             at.level(Variable,1):Sex +
                             at.level(Variable,1):scale(Air.temperature, scale = TRUE) +
                             at.level(Variable,2):Season +
                             at.level(Variable,2):scale(Time, scale = TRUE) +
                             at.level(Variable,2):scale(X..People, scale = TRUE) +
                             at.level(Variable,2):scale(poly(X..Deer, 2), scale = TRUE) + 
                             at.level(Variable,2):scale(BDAY, scale = TRUE) +
                             at.level(Variable,2):scale(poly(Position,2), scale = TRUE) +
                             at.level(Variable,2):scale(Days_in_herd, scale = TRUE) +
                             at.level(Variable,2):scale(Duration, scale = TRUE) +
                             at.level(Variable,2):Sex, 
                           random=~us(Variable):FawnID, rcov=~idh(Variable):units, family= "gaussian", prior=prior,nitt=1050000,thin=500,burnin=50000, data=HR_Autumn,verbose=TRUE, pr = TRUE)
```

We now load our model chain that we used for the main results in the manuscript, after which we give a model summary.

```
R_HR_Autumn_model <- readRDS ("R_HR_Autumn_model.rds")
summary(R_HR_Autumn_model)
```

```
## 
##  Iterations = 50001:1049501
##  Thinning interval  = 500
##  Sample size  = 2000 
## 
##  DIC: 1786.96 
## 
##  G-structure:  ~us(Variable):FawnID
## 
##                                          post.mean l-95% CI u-95% CI eff.samp
## VariableHRend:VariableHRend.FawnID        0.285128  0.13583  0.45918     2000
## VariableScanning:VariableHRend.FawnID     0.004294 -0.07755  0.09101     2000
## VariableHRend:VariableScanning.FawnID     0.004294 -0.07755  0.09101     2000
## VariableScanning:VariableScanning.FawnID  0.159825  0.07330  0.24546     2000
## 
##  R-structure:  ~idh(Variable):units
## 
##                        post.mean l-95% CI u-95% CI eff.samp
## VariableHRend.units       0.5231   0.3832   0.6680     2000
## VariableScanning.units    0.7999   0.6885   0.9336     2000
## 
##  Location effects: Response ~ (Variable - 1) + at.level(Variable, 1):scale(poly(meancap, 2), scale = TRUE) + at.level(Variable, 1):scale(poly(Weight, 2), scale = TRUE) + at.level(Variable, 1):scale(poly(Time, 2), scale = TRUE) + at.level(Variable, 1):Sex + at.level(Variable, 1):scale(Air.temperature, scale = TRUE) + at.level(Variable, 2):Season + at.level(Variable, 2):scale(Time, scale = TRUE) + at.level(Variable, 2):scale(X..People, scale = TRUE) + at.level(Variable, 2):scale(poly(X..Deer, 2), scale = TRUE) + at.level(Variable, 
##     2):scale(BDAY, scale = TRUE) + at.level(Variable, 2):scale(poly(Position, 2), scale = TRUE) + at.level(Variable, 2):scale(Days_in_herd, scale = TRUE) + at.level(Variable, 2):scale(Duration, scale = TRUE) + at.level(Variable, 2):Sex 
## 
##                                                               post.mean  l-95% CI  u-95% CI eff.samp  pMCMC    
## VariableHRend                                                 -0.147208 -0.352332  0.062478     2000  0.156    
## VariableScanning                                              -0.040381 -0.248322  0.188406     2000  0.726    
## at.level(Variable, 1):scale(poly(meancap, 2), scale = TRUE)1   0.097513  0.018123  0.176361     2225  0.015 *  
## at.level(Variable, 1):scale(poly(meancap, 2), scale = TRUE)2  -0.027558 -0.109709  0.059408     2000  0.532    
## at.level(Variable, 1):scale(poly(Weight, 2), scale = TRUE)1    0.187656  0.108724  0.274060     1664 <5e-04 ***
## at.level(Variable, 1):scale(poly(Weight, 2), scale = TRUE)2    0.025429 -0.050222  0.102985     2989  0.536    
## at.level(Variable, 1):scale(poly(Time, 2), scale = TRUE)1      0.119296 -0.006979  0.239317     2147  0.060 .  
## at.level(Variable, 1):scale(poly(Time, 2), scale = TRUE)2      0.010046 -0.106659  0.133861     2000  0.869    
## at.level(Variable, 1):Sexm                                     0.188999 -0.071192  0.468283     2000  0.184    
## at.level(Variable, 1):scale(Air.temperature, scale = TRUE)     0.081352  0.005119  0.161140     2000  0.043 *  
## at.level(Variable, 2):SeasonAutumnwinter2018                   0.308537  0.083667  0.576935     2000  0.017 *  
## at.level(Variable, 2):scale(Time, scale = TRUE)               -0.148646 -0.248255 -0.042413     2000  0.008 ** 
## at.level(Variable, 2):scale(X..People, scale = TRUE)           0.049661 -0.025402  0.120740     1845  0.176    
## at.level(Variable, 2):scale(poly(X..Deer, 2), scale = TRUE)1   0.050872 -0.031459  0.129424     2000  0.213    
## at.level(Variable, 2):scale(poly(X..Deer, 2), scale = TRUE)2   0.080630  0.006115  0.161807     2000  0.050 .  
## at.level(Variable, 2):scale(BDAY, scale = TRUE)               -0.004534 -0.085692  0.084801     2263  0.921    
## at.level(Variable, 2):scale(poly(Position, 2), scale = TRUE)1  0.038387 -0.042139  0.115574     2096  0.359    
## at.level(Variable, 2):scale(poly(Position, 2), scale = TRUE)2  0.088336  0.006291  0.167092     2000  0.029 *  
## at.level(Variable, 2):scale(Days_in_herd, scale = TRUE)       -0.237880 -0.361413 -0.108675     2000 <5e-04 ***
## at.level(Variable, 2):scale(Duration, scale = TRUE)            0.014191 -0.074669  0.112008     2064  0.762    
## Sexm:at.level(Variable, 2)                                    -0.083231 -0.310150  0.141457     2000  0.480    
## ---
## Signif. codes:  0 '***' 0.001 '**' 0.01 '*' 0.05 '.' 0.1 ' ' 1
```

We then check for chain convergence. We have ran separate chains which we load here, but feel free to run new chains of your own. instead.

```
mean(summary(R_HR_Autumn_model)$Gcovariances[,4])
```

```
## [1] 2000
```

```
mean(abs(autocorr.diag(R_HR_Autumn_model$VCV[,1:4], lag=c(1))))
```

```
## [1] 0.01033894
```

```
R_HR_Autumn_model2 <- readRDS ("R_HR_Autumn_model2.rds")
R_HR_Autumn_model3 <- readRDS ("R_HR_Autumn_model3.rds")
R_HR_Autumn_model4 <- readRDS ("R_HR_Autumn_model4.rds")

diag = gelman.diag(mcmc.list(R_HR_Autumn_model$Sol, R_HR_Autumn_model2$Sol, R_HR_Autumn_model3$Sol, R_HR_Autumn_model4$Sol ))
diag$mpsrf
```

```
## [1] 1.059521
```

Everything seems okay. We have finalized this model.

### A.4) Latency-scanning autumn model

We provide the code for the full model below.

```
Lat_Autumn<- MCMCglmm(Response~(Variable-1)+
                        at.level(Variable,1):scale(meancap, scale = TRUE) +
                        at.level(Variable,1):scale(poly(Weight,2), scale = TRUE) +
                        at.level(Variable,1):Year +
                        at.level(Variable,1):scale(Capture, scale = FALSE) +
                        at.level(Variable,2):Season +
                        at.level(Variable,2):scale(poly(Time,2), scale = TRUE) +
                        at.level(Variable,2):scale(poly(X..People,2), scale = TRUE) +
                        at.level(Variable,2):scale(poly(X..Deer, 2), scale = TRUE) + 
                        at.level(Variable,2):scale(poly(BDAY,2), scale = TRUE) +
                        at.level(Variable,2):scale(poly(Days_in_herd,2), scale = TRUE) +
                        at.level(Variable,2):scale(poly(Duration,2), scale = TRUE) +
                        at.level(Variable,2):Sex, 
                      random=~us(Variable):FawnID, rcov=~idh(Variable):units, family= "gaussian", prior=prior,nitt=1050000,thin=500,burnin=50000, data=Latency_Autumn,verbose=TRUE, pr = TRUE)
```

We have saved the model chain we used. We will load that model now and inspect the output.

```
Lat_Autumn <- readRDS ("Lat_Autumn_model.rds")
summary(Lat_Autumn)
```

```
## 
##  Iterations = 50001:1049501
##  Thinning interval  = 500
##  Sample size  = 2000 
## 
##  DIC: 1789.613 
## 
##  G-structure:  ~us(Variable):FawnID
## 
##                                          post.mean l-95% CI u-95% CI eff.samp
## VariableLatency:VariableLatency.FawnID     0.27135  0.13591 0.443754     1885
## VariableScanning:VariableLatency.FawnID   -0.07218 -0.15054 0.009506     2000
## VariableLatency:VariableScanning.FawnID   -0.07218 -0.15054 0.009506     2000
## VariableScanning:VariableScanning.FawnID   0.16802  0.08352 0.259057     2000
## 
##  R-structure:  ~idh(Variable):units
## 
##                        post.mean l-95% CI u-95% CI eff.samp
## VariableLatency.units     0.5564   0.4118   0.6896     2000
## VariableScanning.units    0.7941   0.6799   0.9208     2000
## 
##  Location effects: Response ~ (Variable - 1) + at.level(Variable, 1):scale(meancap, scale = TRUE) + at.level(Variable, 1):scale(poly(Weight, 2), scale = TRUE) + at.level(Variable, 1):Year + at.level(Variable, 1):scale(Capture, scale = FALSE) + at.level(Variable, 2):Season + at.level(Variable, 2):scale(poly(Time, 2), scale = TRUE) + at.level(Variable, 2):scale(poly(X..People, 2), scale = TRUE) + at.level(Variable, 2):scale(poly(X..Deer, 2), scale = TRUE) + at.level(Variable, 2):scale(poly(BDAY, 2), scale = TRUE) + at.level(Variable, 
##     2):scale(poly(Position, 2), scale = TRUE) + at.level(Variable, 2):scale(poly(Days_in_herd, 2), scale = TRUE) + at.level(Variable, 2):scale(poly(Duration, 2), scale = TRUE) + at.level(Variable, 2):Sex 
## 
##                                                                   post.mean  l-95% CI  u-95% CI eff.samp  pMCMC    
## VariableLatency                                                   -0.966779 -1.538349 -0.376654     2000 <5e-04 ***
## VariableScanning                                                  -0.032302 -0.266079  0.193634     2000  0.790    
## at.level(Variable, 1):scale(meancap, scale = TRUE)                -0.133300 -0.218974 -0.057279     2000  0.002 ** 
## at.level(Variable, 1):scale(poly(Weight, 2), scale = TRUE)1       -0.084850 -0.170301  0.007792     1810  0.070 .  
## at.level(Variable, 1):scale(poly(Weight, 2), scale = TRUE)2        0.063460 -0.014259  0.137130     1654  0.107    
## at.level(Variable, 1):Year2019                                     0.133777 -0.152694  0.373701     2000  0.347    
## at.level(Variable, 1):scale(Capture, scale = FALSE)               -0.248207 -0.405822 -0.107254     2145  0.002 ** 
## at.level(Variable, 2):SeasonAutumnwinter2018                       0.273308  0.017184  0.534167     2000  0.035 *  
## at.level(Variable, 2):scale(poly(Time, 2), scale = TRUE)1         -0.144513 -0.248092 -0.034465     2000  0.007 ** 
## at.level(Variable, 2):scale(poly(Time, 2), scale = TRUE)2          0.049595 -0.038851  0.138087     2000  0.278    
## at.level(Variable, 2):scale(poly(X..People, 2), scale = TRUE)1     0.042662 -0.029935  0.119242     1760  0.275    
## at.level(Variable, 2):scale(poly(X..People, 2), scale = TRUE)2    -0.015187 -0.095265  0.062000     2416  0.699    
## at.level(Variable, 2):scale(poly(X..Deer, 2), scale = TRUE)1       0.045563 -0.035403  0.131362     2147  0.289    
## at.level(Variable, 2):scale(poly(X..Deer, 2), scale = TRUE)2       0.075446 -0.013631  0.156200     2000  0.087 .  
## at.level(Variable, 2):scale(poly(BDAY, 2), scale = TRUE)1         -0.012662 -0.102690  0.071035     1958  0.785    
## at.level(Variable, 2):scale(poly(BDAY, 2), scale = TRUE)2         -0.068486 -0.166525  0.030361     2000  0.177    
## at.level(Variable, 2):scale(poly(Position, 2), scale = TRUE)1      0.039488 -0.041463  0.121679     3176  0.336    
## at.level(Variable, 2):scale(poly(Position, 2), scale = TRUE)2      0.078835  0.001873  0.167949     2000  0.058 .  
## at.level(Variable, 2):scale(poly(Days_in_herd, 2), scale = TRUE)1 -0.248534 -0.381120 -0.114898     2462 <5e-04 ***
## at.level(Variable, 2):scale(poly(Days_in_herd, 2), scale = TRUE)2 -0.001154 -0.135371  0.120403     2000  0.978    
## at.level(Variable, 2):scale(poly(Duration, 2), scale = TRUE)1      0.048285 -0.053861  0.158985     1864  0.386    
## at.level(Variable, 2):scale(poly(Duration, 2), scale = TRUE)2     -0.050578 -0.149693  0.041075     2000  0.313    
## at.level(Variable, 2):Sexm                                        -0.061583 -0.292534  0.177730     2000  0.581    
## ---
## Signif. codes:  0 '***' 0.001 '**' 0.01 '*' 0.05 '.' 0.1 ' ' 1
```

Based on this full model, we now create our simplified final models. We do so by removing the quadratic effects which have a p > 0.1. Those are “Duration”, “Days in herd”, “BDAY”, “X..People”, “Time”. This model is now as following:

```
R_Lat_Autumn<- MCMCglmm(Response~(Variable-1)+
                        at.level(Variable,1):scale(meancap, scale = TRUE) +
                        at.level(Variable,1):scale(poly(Weight,2), scale = TRUE) +
                        at.level(Variable,1):Year +
                        at.level(Variable,1):scale(Capture, scale = FALSE) +
                        at.level(Variable,2):Season +
                        at.level(Variable,2):scale(Time, scale = TRUE) +
                        at.level(Variable,2):scale(X..People, scale = TRUE) +
                        at.level(Variable,2):scale(poly(X..Deer, 2), scale = TRUE) + 
                        at.level(Variable,2):scale(BDAY, scale = TRUE) +
                        at.level(Variable,2):scale(poly(Position,2), scale = TRUE) +
                        at.level(Variable,2):scale(Days_in_herd, scale = TRUE) +
                        at.level(Variable,2):scale(Duration, scale = TRUE) +
                        at.level(Variable,2):Sex, 
                      random=~us(Variable):FawnID, rcov=~idh(Variable):units, family= "gaussian", prior=prior,nitt=1050000,thin=500,burnin=50000, data=Latency_Autumn,verbose=TRUE, pr = TRUE)
```

We now load our model chain that we used for the main results in the manuscript, after which we give a model summary.

```
R_Lat_Autumn <- readRDS ("R_Lat_Autumn_model.rds")
summary(R_Lat_Autumn)
```

```
## 
##  Iterations = 50001:1049501
##  Thinning interval  = 500
##  Sample size  = 2000 
## 
##  DIC: 1784.551 
## 
##  G-structure:  ~us(Variable):FawnID
## 
##                                          post.mean l-95% CI u-95% CI eff.samp
## VariableLatency:VariableLatency.FawnID     0.27428  0.13032 0.427075     2000
## VariableScanning:VariableLatency.FawnID   -0.07592 -0.15851 0.009591     1899
## VariableLatency:VariableScanning.FawnID   -0.07592 -0.15851 0.009591     1899
## VariableScanning:VariableScanning.FawnID   0.16909  0.08716 0.263584     2013
## 
##  R-structure:  ~idh(Variable):units
## 
##                        post.mean l-95% CI u-95% CI eff.samp
## VariableLatency.units     0.5559   0.4227   0.6957     2000
## VariableScanning.units    0.7932   0.6723   0.9108     2000
## 
##  Location effects: Response ~ (Variable - 1) + at.level(Variable, 1):scale(meancap, scale = TRUE) + at.level(Variable, 1):scale(poly(Weight, 2), scale = TRUE) + at.level(Variable, 1):Year + at.level(Variable, 1):scale(Capture, scale = FALSE) + at.level(Variable, 2):Season + at.level(Variable, 2):scale(Time, scale = TRUE) + at.level(Variable, 2):scale(X..People, scale = TRUE) + at.level(Variable, 2):scale(poly(X..Deer, 2), scale = TRUE) + at.level(Variable, 2):scale(BDAY, scale = TRUE) + at.level(Variable, 2):scale(poly(Position, 
##     2), scale = TRUE) + at.level(Variable, 2):scale(Days_in_herd, scale = TRUE) + at.level(Variable, 2):scale(Duration, scale = TRUE) + at.level(Variable, 2):Sex 
## 
##                                                               post.mean  l-95% CI  u-95% CI eff.samp  pMCMC    
## VariableLatency                                               -0.977307 -1.592009 -0.414919     2141 <5e-04 ***
## VariableScanning                                              -0.034419 -0.254371  0.180655     2420  0.777    
## at.level(Variable, 1):scale(meancap, scale = TRUE)            -0.133305 -0.217209 -0.054290     2000  0.002 ** 
## at.level(Variable, 1):scale(poly(Weight, 2), scale = TRUE)1   -0.082647 -0.167157  0.011331     2000  0.080 .  
## at.level(Variable, 1):scale(poly(Weight, 2), scale = TRUE)2    0.062537 -0.011463  0.142147     2000  0.101    
## at.level(Variable, 1):Year2019                                 0.132414 -0.120857  0.409725     2391  0.316    
## at.level(Variable, 1):scale(Capture, scale = FALSE)           -0.252071 -0.401271 -0.102370     2174  0.001 ***
## at.level(Variable, 2):SeasonAutumnwinter2018                   0.303596  0.054123  0.532255     2000  0.013 *  
## at.level(Variable, 2):scale(Time, scale = TRUE)               -0.144453 -0.251891 -0.045745     2156  0.011 *  
## at.level(Variable, 2):scale(X..People, scale = TRUE)           0.052149 -0.019693  0.130799     2000  0.166    
## at.level(Variable, 2):scale(poly(X..Deer, 2), scale = TRUE)1   0.054858 -0.027186  0.134217     1828  0.178    
## at.level(Variable, 2):scale(poly(X..Deer, 2), scale = TRUE)2   0.073553 -0.006384  0.150774     2147  0.076 .  
## at.level(Variable, 2):scale(BDAY, scale = TRUE)               -0.010852 -0.093081  0.081330     2133  0.821    
## at.level(Variable, 2):scale(poly(Position, 2), scale = TRUE)1  0.037269 -0.040858  0.122674     2000  0.374    
## at.level(Variable, 2):scale(poly(Position, 2), scale = TRUE)2  0.089323  0.002254  0.175287     2000  0.045 *  
## at.level(Variable, 2):scale(Days_in_herd, scale = TRUE)       -0.239002 -0.365949 -0.120363     2000 <5e-04 ***
## at.level(Variable, 2):scale(Duration, scale = TRUE)            0.020357 -0.075507  0.109121     2000  0.677    
## at.level(Variable, 2):Sexm                                    -0.091501 -0.331911  0.123599     2000  0.432    
## ---
## Signif. codes:  0 '***' 0.001 '**' 0.01 '*' 0.05 '.' 0.1 ' ' 1
```

We then check for chain convergence. We have ran separate chains which we load here, but feel free to run new chains of your own. instead.

```
mean(summary(R_Lat_Autumn)$Gcovariances[,4])
```

```
## [1] 1952.935
```

```
mean(abs(autocorr.diag(R_Lat_Autumn$VCV[,1:4], lag=c(1))))
```

```
## [1] 0.0171133
```

```
R_Lat_Autumn2 <- readRDS ("R_Lat_Autumn_model2.rds")
R_Lat_Autumn3 <- readRDS ("R_Lat_Autumn_model3.rds")
R_Lat_Autumn4 <- readRDS ("R_Lat_Autumn_model4.rds")

diag = gelman.diag(mcmc.list(R_Lat_Autumn$Sol, R_Lat_Autumn2$Sol, R_Lat_Autumn3$Sol, R_Lat_Autumn4$Sol ))
diag$mpsrf
```

```
## [1] 1.055915
```

Everything seems okay. We have finalized this model. We have now finalized the models.

# Section B: Results

### Repeatability

We’ll start with the repeatability estimates of the neonate capture traits. For these, we will use the autumn models since they have a bigger sample size.

```
# Heart rate 
Rpt_HR <- R_HR_Autumn_model$VCV[,"VariableHRend:VariableHRend.FawnID"]/(
  R_HR_Autumn_model$VCV[,"VariableHRend:VariableHRend.FawnID"] +
    R_HR_Autumn_model$VCV[,"VariableHRend.units"])

plot(Rpt_HR)
```

```
mean(Rpt_HR)
```

```
## [1] 0.3500953
```

```
HPDinterval(Rpt_HR)
```

```
##          lower     upper
## var1 0.1754932 0.5154668
## attr(,"Probability")
## [1] 0.95
```

```
# Latency to leave
Rpt_Lat <- R_Lat_Autumn$VCV[,"VariableLatency:VariableLatency.FawnID"]/(
  R_Lat_Autumn$VCV[,"VariableLatency:VariableLatency.FawnID"] +
    R_Lat_Autumn$VCV[,"VariableLatency.units"])

plot(Rpt_Lat)
```

```
mean(Rpt_Lat)
```

```
## [1] 0.3280923
```

```
HPDinterval(Rpt_Lat)
```

```
##          lower     upper
## var1 0.1737115 0.4756148
## attr(,"Probability")
## [1] 0.95
```

We then continue with the repeatability of scanning. Since we have run two summer models and two autumn models, we’ll compute them from both, for transparency purposes. The estimates are near identical between the models within each season.

```
# Scanning in summer, from the heart rate model.
Rpt_Summer_Scanning <- R_HR_Summer_model$VCV[,"VariableScanning:VariableScanning.FawnID"]/(
  R_HR_Summer_model$VCV[,"VariableScanning:VariableScanning.FawnID"] +
    R_HR_Summer_model$VCV[,"VariableScanning.units"])

plot(Rpt_Summer_Scanning)
```

```
mean(Rpt_Summer_Scanning)
```

```
## [1] 0.1171443
```

```
HPDinterval(Rpt_Summer_Scanning)
```

```
##           lower     upper
## var1 0.05485504 0.1786637
## attr(,"Probability")
## [1] 0.95
```

```
# And also the estimates from the latency summer model. 
Rpt_Summer_Scanning <- R_Lat_Summer$VCV[,"VariableScanning:VariableScanning.FawnID"]/(
  R_Lat_Summer$VCV[,"VariableScanning:VariableScanning.FawnID"] +
    R_Lat_Summer$VCV[,"VariableScanning.units"])

mean(Rpt_Summer_Scanning)
```

```
## [1] 0.1159375
```

```
HPDinterval(Rpt_Summer_Scanning)
```

```
##           lower     upper
## var1 0.06113759 0.1772158
## attr(,"Probability")
## [1] 0.95
```

```
# Scanning in autumn, from the heart rate model.
Rpt_Autumn_Scanning <- R_HR_Autumn_model$VCV[,"VariableScanning:VariableScanning.FawnID"]/(
  R_HR_Autumn_model$VCV[,"VariableScanning:VariableScanning.FawnID"] +
    R_HR_Autumn_model$VCV[,"VariableScanning.units"])

plot(Rpt_Autumn_Scanning)
```

```
mean(Rpt_Autumn_Scanning)
```

```
## [1] 0.1658933
```

```
HPDinterval(Rpt_Autumn_Scanning)
```

```
##           lower    upper
## var1 0.08507821 0.248884
## attr(,"Probability")
## [1] 0.95
```

```
# And also the estimates from the latency Autumn model. 
Rpt_Autumn_Scanning <- R_Lat_Autumn$VCV[,"VariableScanning:VariableScanning.FawnID"]/(
  R_Lat_Autumn$VCV[,"VariableScanning:VariableScanning.FawnID"] +
    R_Lat_Autumn$VCV[,"VariableScanning.units"])

mean(Rpt_Autumn_Scanning)
```

```
## [1] 0.1748498
```

```
HPDinterval(Rpt_Autumn_Scanning)
```

```
##           lower     upper
## var1 0.09722146 0.2588851
## attr(,"Probability")
## [1] 0.95
```

### Covariation at the among-individual level

Finally, we will compute the covariations at the among-individual level. We’ll do that for all four models, which is the last step in this analysis.

```
# Covariation between heart rate and summer scanning 
Covar_HR_Summer_Scanning <- R_HR_Summer_model$VCV[,"VariableScanning:VariableHRend.FawnID"]/
  (sqrt(R_HR_Summer_model$VCV[,"VariableScanning:VariableScanning.FawnID"])*
     sqrt(R_HR_Summer_model$VCV[,"VariableHRend:VariableHRend.FawnID"]))

mean(Covar_HR_Summer_Scanning)
```

```
## [1] -0.1627701
```

```
HPDinterval(Covar_HR_Summer_Scanning)
```

```
##           lower     upper
## var1 -0.5207829 0.2249101
## attr(,"Probability")
## [1] 0.95
```

```
plot(Covar_HR_Summer_Scanning)
```

```
# Covariation between latency and summer scanning
Covar_Latency_Summer_Scanning <- R_Lat_Summer$VCV[,"VariableScanning:VariableLatency.FawnID"]/
  (sqrt(R_Lat_Summer$VCV[,"VariableScanning:VariableScanning.FawnID"])*
     sqrt(R_Lat_Summer$VCV[,"VariableLatency:VariableLatency.FawnID"]))

mean(Covar_Latency_Summer_Scanning)
```

```
## [1] -0.03469059
```

```
HPDinterval(Covar_Latency_Summer_Scanning)
```

```
##           lower     upper
## var1 -0.3942216 0.3740056
## attr(,"Probability")
## [1] 0.95
```

```
plot(Covar_Latency_Summer_Scanning)
```

```
# Covariation between heart rate and autumn scanning 
Covar_HR_Autumn_Scanning <- R_HR_Autumn_model$VCV[,"VariableScanning:VariableHRend.FawnID"]/
  (sqrt(R_HR_Autumn_model$VCV[,"VariableScanning:VariableScanning.FawnID"])*
     sqrt(R_HR_Autumn_model$VCV[,"VariableHRend:VariableHRend.FawnID"]))

mean(Covar_HR_Autumn_Scanning)
```

```
## [1] 0.02021743
```

```
HPDinterval(Covar_HR_Autumn_Scanning)
```

```
##           lower     upper
## var1 -0.3437866 0.4264991
## attr(,"Probability")
## [1] 0.95
```

```
plot(Covar_HR_Autumn_Scanning)
```

```
# Covariation between latency and autumn scanning
Covar_Latency_Autumn_Scanning <- R_Lat_Autumn$VCV[,"VariableScanning:VariableLatency.FawnID"]/
  (sqrt(R_Lat_Autumn$VCV[,"VariableScanning:VariableScanning.FawnID"])*
     sqrt(R_Lat_Autumn$VCV[,"VariableLatency:VariableLatency.FawnID"]))

mean(Covar_Latency_Autumn_Scanning)
```

```
## [1] -0.3526817
```

```
HPDinterval(Covar_Latency_Autumn_Scanning)
```

```
##           lower       upper
## var1 -0.6739526 -0.01762136
## attr(,"Probability")
## [1] 0.95
```

```
plot(Covar_Latency_Autumn_Scanning)
```
